# Regulation of Endocytic Clathrin Dynamics by Cargo Ubiquitination

**DOI:** 10.1016/j.devcel.2012.08.003

**Published:** 2012-09-11

**Authors:** Anastasia G. Henry, James N. Hislop, Joe Grove, Kurt Thorn, Mark Marsh, Mark von Zastrow

**Affiliations:** 1 Program in Cell Biology, University of California, San Francisco, San Francisco, CA 94158, USA; 2 Department of Psychiatry, University of California, San Francisco, San Francisco, CA 94158, USA; 3 Department of Cellular and Molecular Pharmacology, University of California, San Francisco, San Francisco, CA 94158, USA; 4 Department of Biochemistry and Biophysics, University of California, San Francisco, San Francisco, CA 94158, USA; 5 MRC Laboratory for Molecular Cell Biology, University College London, London WC1E 6BT, UK

## Abstract

Some endocytic cargoes control clathrin-coated pit (CCP) maturation, but it is not known how such regulation is communicated. We found that μ-opioid neuropeptide receptors signal to their enclosing CCPs by ubiquitination. Nonubiquitinated receptors delay CCPs at an intermediate stage of maturation, after clathrin lattice assembly is complete but before membrane scission. Receptor ubiquitination relieves this inhibition, effectively triggering CCP scission and producing a receptor-containing endocytic vesicle. The ubiquitin modification that conveys this endocytosis-promoting signal is added to the receptor’s first cytoplasmic loop, catalyzed by the Smurf2 ubiquitin ligase, and coordinated with activation-dependent receptor phosphorylation and clustering through Smurf2 recruitment by the endocytic adaptor beta-arrestin. Epsin1 detects the signal at the CCP and is required for ubiquitin-promoted scission. This cargo-to-coat communication system mediates a biochemical checkpoint that ensures appropriate receptor ubiquitination for later trafficking, and it controls specific receptor loading into CCPs by sensing when a sufficient quorum is reached.

**Video Abstract:**

## Highlights

► μ-opioid receptor (MOR) ubiquitination by the E3 ligase Smurf2 triggers endocytosis ► Nonubiquitinated MORs prolong the surface lifetime of clathrin-coated pits (CCPs) ► Kinetic control of CCPs assures receptor ubiquitination for postendocytic traffic ► Kinetic control of CCPs also helps determine endocytic cargo load

## Teaser

Some endocytic cargoes regulate clathrin-coated pit (CCP) maturation, but how such regulation is communicated remains unknown. Henry et al. show that μ-opioid receptors signal to their enclosing CCPs by ubiquitination, allowing cargo-mediated control of CCP dynamics. The authors suggest that this modulates the receptor load of CCPs and, later, receptor trafficking.

## Introduction

Clathrin-coated pits (CCPs) mediate endocytosis of diverse membrane cargoes and are essential for numerous cellular processes, from the uptake of nutrients to the regulation of receptor-mediated signaling. Clathrin-dependent endocytosis involves a precisely orchestrated series of events, subject to regulation at the level of the conserved endocytic machinery and accessory proteins (Perrais and Merrifield, 2005; Toret and Drubin, 2006; Traub, 2009). The clathrin-containing lattice promotes membrane deformation and captures cargoes through interaction with various adaptors or CLASPs (Brodsky et al., 2001; Farsad and De Camilli, 2003; Itoh and De Camilli, 2006; Maldonado-Báez and Wendland, 2006; McMahon and Gallop, 2005; Traub, 2009). CCPs then undergo membrane scission, regulated in animal cells by dynamin (Taylor et al., 2012), producing endocytic vesicles. While CCP formation and function are exquisitely regulated (Kirchhausen, 2009; Taylor et al., 2011; Weinberg and Drubin, 2011), membrane cargoes packaged in the CCP have been generally viewed as inert passengers. However, it is increasingly clear that some cargoes can influence the initial formation of CCPs or their subsequent maturation (Ehrlich et al., 2004; Liu et al., 2010; Loerke et al., 2009; Mettlen et al., 2010; Puthenveedu and von Zastrow, 2006; Rust et al., 2004). An interesting, and largely unresolved, question is how such cargo-mediated control is communicated.

Cargo control is particularly interesting for signal-transducing receptors that are regulated by CCP-dependent endocytosis. There is already evidence for feedback control of the endocytic pathway at a global level by downstream signaling effectors (Le Roy and Wrana, 2005; Sorkin and von Zastrow, 2009). It is not known if activated receptors can also signal locally to the endocytic machinery, effectively exerting direct control over their enclosing CCPs. Two properties of seven-transmembrane receptors (7TMRs), the largest known family of signaling receptors, make them prime candidates for mediating such local modulation. First, many 7TMRs form oligomeric complexes and cluster nonuniformly in CCPs following ligand-induced activation, forming a 7TMR-enriched CCP subset under conditions of endogenous or near-endogenous levels of receptor and adaptor expression (Cao et al., 1998; Kasai et al., 2011; Khelashvili et al., 2010; Mundell et al., 2006; Puthenveedu and von Zastrow, 2006). Second, some 7TMRs are already known to engage actin-binding proteins after clustering in CCPs, thereby locally prolonging the surface residence time of 7TMR-containing CCPs before they internalize (Puthenveedu and von Zastrow, 2006). In addition to this apparently passive cargo-based prolongation of CCP maturation, might some receptors locally convey an active endocytosis-promoting signal? Here, we show that this is indeed the case, and identify an ubiquitin-dependent signaling mechanism that regulates endocytosis by locally controlling the surface lifetime of receptor-containing CCPs.

## Results

### Ubiquitination Controls the Rate of MOR Endocytosis by CCPs

**Figure 1:**
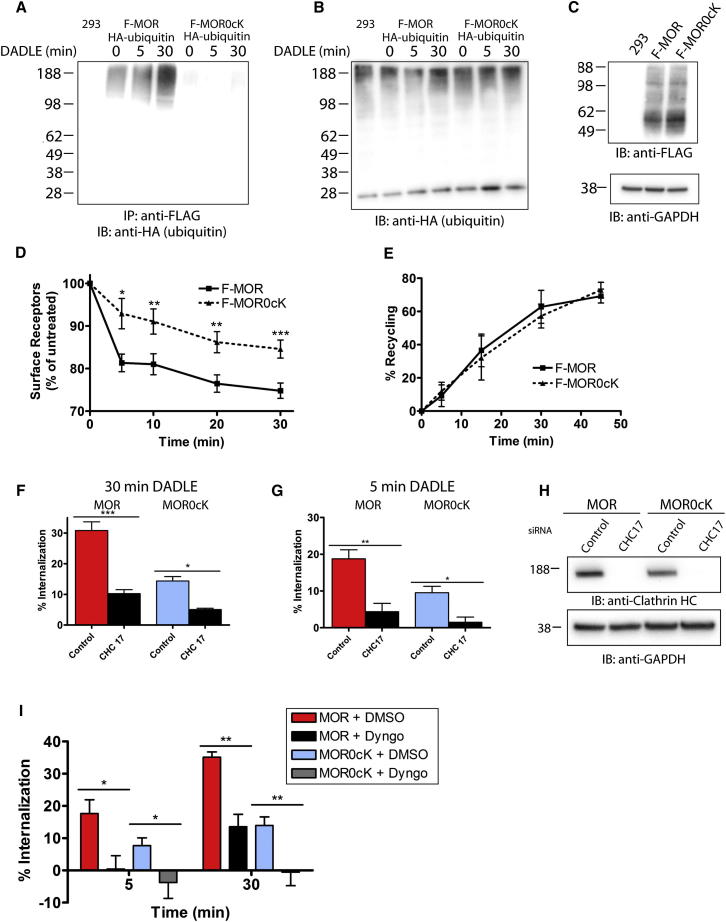
Receptor Ubiquitination Is Required for Efficient Endocytosis of MOR (A) The ubiquitination status of wild-type or lysyl mutant MORs (F-MOR or F-MOR0cK, respectively) was examined after treatment with 10 μM DADLE before extraction and immunoprecipitation with FLAG antibody. Shown is a representative anti-HA blot of untransfected cells (293), F-MOR- (left), and F-MOR0cK-expressing cells (right). (B and C) Lysates from cells in (A) probed with HA antibody (B) or FLAG and GAPDH antibody (C) to control for equal loading and expression. (D) Time course of endocytosis for F-MOR and F-MOR0cK receptors after DADLE treatment for the indicated time points. The amount of surface receptors was measured using flow cytometry; n = 4. (E) Time course of receptor recycling after 30 min DADLE treatment, agonist washout, and treatment with 10 μM of opioid antagonist naloxone for the indicated times. Shown is the recycling efficiency; n = 3. (F and G) Flow cytometric analysis of receptor internalization after clathrin knockdown and 30 min (F) or 5 min (G) DADLE application; n = 3 and n ≥ 4, respectively. (H) To verify clathrin knockdown, immunoblots detecting endogenous clathrin heavy chain were performed, and equal loading was confirmed by immunoblotting for GAPDH. (I) Flow cytometric analysis of receptor internalization after treatment with vehicle or the dynamin inhibitor, DYNGO, for 15 min followed by treatment with DADLE for 5 or 30 min; n = 3. Error bars indicate standard error of the mean (SEM). p values: one- or two-way ANOVA, Bonferroni multiple comparison test; ^∗^p < 0.05; ^∗∗^p < 0.01; ^∗∗∗^p < 0.001. See also Figure S2.

FLAG-tagged mu opioid receptors (F-MORs) were basally ubiquitinated at a low level, and receptor ubiquitination increased over a period of minutes in response to the opioid receptor agonist DADLE (Figure 1A, left lanes). Mutating all cytoplasmic lysine residues (F-MOR0cK) prevented both basal and agonist-induced receptor ubiquitination (Figures 1A, right lanes; loading controls in Figures 1B and 1C). Blocking F-MOR internalization using K44E mutant dynamin or the dynamin inhibitor Dyngo-4a did not (data not shown), indicating that MOR ubiquitination can occur prior to endocytosis. Lysine mutation inhibited agonist-induced internalization of receptors but had no effect on the ability of receptors to recycle back to the cell surface (Figures 1D and 1E), and agonist-induced endocytosis of receptors remained both clathrin and dynamin-dependent (Figures 1F–1I). Thus, preventing MOR ubiquitination alters the rate but not mechanism of agonist-induced endocytosis.

### Ubiquitination Regulates MOR Endocytosis after Receptors Cluster in CCPs

**Figure 2:**
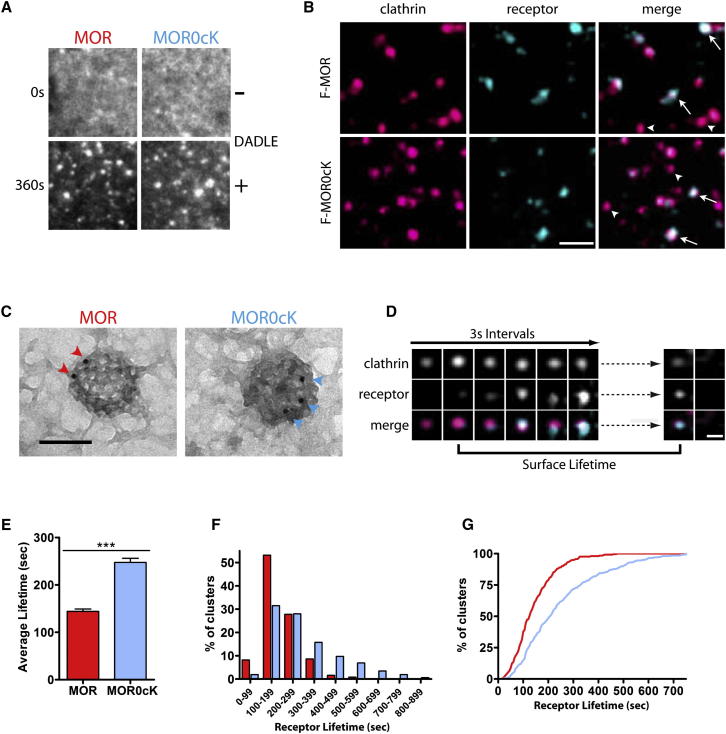
MOR Ubiquitination Is Not Required for Receptor Entry into CCPs and Selectively Controls Surface Lifetime of Receptors after Clustering (A) TIRF live-cell imaging of cells expressing F-MOR or F-MOR0cK before and after 10 min treatment with 10 μM DADLE. (B) Cells expressing DsRed-tagged clathrin light chain (shown in fushia) and either F-MOR or F-MOR0cK (shown in teal) were imaged live. Shown is a representative image 10 min after DADLE application. Arrowheads indicate CCPs that lack receptor while arrows indicate CCPs that contain receptor; scale bar = 1 μm. (C) Example immunoelectron micrographs of CCPs containing F-MORs (left) or F-MOR0cKs (right) labeled with FLAG antibody and protein A conjugated to 10 nm gold particles treated with agonist for 2 min. Samples were prepared as described in Experimental Procedures; scale bar = 100 nm. (D) Representative time lapse series showing MORs cluster into preexisting CCPs. Cells were treated with agonist while imaging using TIRF-M. Frames are 3 s apart; scale bar = 500 nm. (E) The average lifetimes that F-MOR and F-MOR0cK clusters remain on the cell surface before undergoing endocytic scission; F-MOR n = 256 clusters, ten cells; F-MOR0cK n = 318 clusters, 12 cells. (F and G) Frequency distribution (F) and cumulative probability (G) analysis of MOR (red) and MOR0cK (light blue) cluster lifetimes. Error bars indicate standard error of the mean (SEM). p values: Student’s t test; ^∗∗∗^p < 0.001. See also Figure S1 and Movie S1.

Clathrin-dependent endocytosis of 7TMRs in animal cells is initiated by arrestin-promoted clustering of activated receptors into preexisting CCPs (Santini et al., 2002). Because ubiquitin interacts with various endocytic adaptors (Hicke and Dunn, 2003; Shih et al., 2002; Torrisi et al., 1999; Toshima et al., 2009), we expected lysine mutation to impair the clustering step but this was not the case. Total internal reflection fluorescence microscopy (TIR-FM) indicated that both F-MORs and F-MOR0cKs were diffusely distributed in the plasma membrane in the absence of agonist and clustered into diffraction-limited spots after agonist application (Figure 2A), as verified in our optical system by imaging 50 nm fluorescent beads (data not shown). The time required for clustering after agonist addition was similar for MORs and MOR0cKs (Figure S1A available online). Robust clustering was also observed using a covalent rather than antibody-based labeling strategy (Figure S1C) based on fusion of a pH-sensitive green fluorescent protein (GFP) variant (superecliptic pHluorin, or SpH) whose fluorescence is quenched in endosomes (Miesenböck et al., 1998).

Diffraction-limited receptor spots observed in agonist-exposed cells were immobile and colocalized with clathrin light chain (Figure 2B, arrows), supporting their identification as receptor-containing CCPs (Puthenveedu and von Zastrow, 2006). TIR-FM analysis was restricted to the ventral plasma membrane, but immunoelectron microscopy revealed clustering of both MORs and MOR0cKs into morphologically characteristic CCPs in the dorsal plasma membrane (Figure 2C). CCPs containing both F-MOR and F-MOR0cK were productive because the clathrin coat disappeared from the TIR-FM imaging field concomitantly with receptor removal from the plasma membrane (Figure 2D; Movie S1). Further, discrete endocytic events, when summed over the imaged surface and time, were sufficient to account for net receptor loss determined by integrated receptor fluorescence (Figures S2A and S2B). We also verified that disappearance of both MOR and MOR0cK clusters was preceded by a characteristic burst of GFP-tagged dynamin-2 accumulation (not shown). Together these results indicate that both wild-type MOR and ubiquitination-defective MOR0cK undergo regulated endocytosis via the conserved CCP pathway, involving agonist-induced clustering of receptors into CCPs followed by characteristic dynamin-dependent endocytic scission.

CCPs typically undergo scission within seconds after coat assembly is complete (Ehrlich et al., 2004; Kaksonen et al., 2005; Merrifield et al., 2002; Taylor et al., 2011) but CCPs containing MORs, in contrast, were consistently found to linger in the plasma membrane. We estimated for each CCP a receptor “surface lifetime,” defined as the interval between the initial appearance of a diffraction-limited receptor spot (the frame in which its surface intensity visibly exceeded the plasma membrane surround) and its later abrupt disappearance from the TIR-FM illumination field (indicating endocytic scission). Lysine mutation of MORs markedly extended the mean surface lifetime of receptor-containing CCPs (Figure 2E) and right-shifted the respective frequency (Figure 2F) and cumulative probability (Figure 2G) distributions of events. We further verified this effect using receptors labeled covalently with SpH, to specifically visualize surface receptors without the potential complications of bound antibody (Figures S2C–S2E). Together, these results reveal an unanticipated role of 7TMR ubiquitination in limiting the surface lifetime of CCPs that mediate their endocytosis.

### Kinetic Control Is Conferred by Agonist-Induced Ubiquitination of the Receptor’s First Cytoplasmic Loop

**Figure 3:**
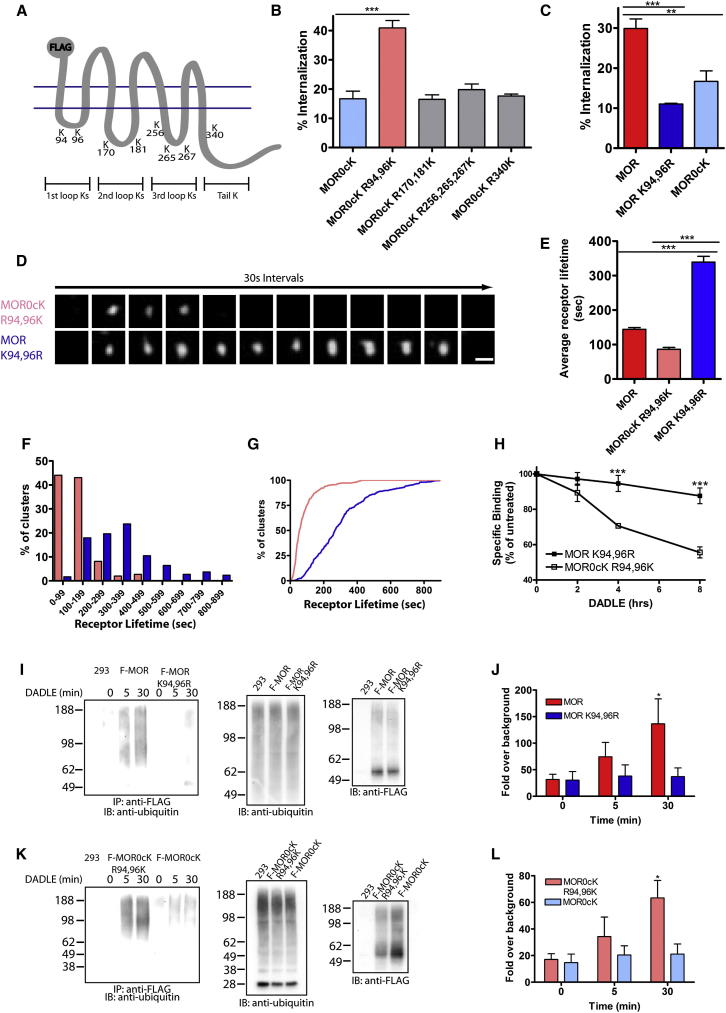
Lysine 94 and 96 in the First Intracellular Loop of MOR Control the Surface Lifetime of Receptors and Are the Major Sites of Agonist-Induced MOR Ubiquitination (A) Schematic of MOR indicating the positions of the eight cytoplasmic lysine residues. (B) Flow cytometric analysis of the internalization of mutant receptors with no cytoplasmic lysine residues (MOR0cK) and with the selective return of lysine residues in the different intracellular domains of the receptor corresponding to the sites shown in (A). Cells were treated with 10 μM DADLE for 30 min; n = 3. (C) The percentage of receptor internalization was measured as in (B) in cells expressing wild-type MOR, MOR K94,96R, and MOR0cK; n = 3. (D) Representative image sequence of clusters of mutant receptors with only the first intracellular loop lysines present (MOR0cK R94,96K) or only missing these sites (MOR K94,96R). Cells were treated with agonist while imaging using TIRF-M. Frames are 30 s apart; scale bar = 500 nm. (E) The average surface lifetimes of MOR, MOR0cK R94,96K, and MOR K94,96R clusters after agonist treatment; MOR n = 256 clusters, ten cells; MOR0cK R94,96K n = 296 clusters, 11 cells; and MOR K94,96R n = 274 clusters, ten cells. (F and G) Frequency distribution (F) and cumulative probability analysis (G) of MOR0cK R94,96K (pink) and MOR K94,96R (blue) cluster lifetimes. (H) Cells expressing F- MOR K94,96R or F-MOR0cK R94,96K were incubated for the indicated times with agonist before undergoing freeze-thaw and radioligand binding with [^3^H]diprenorphine. The specific binding values, expressed as a percentage of binding in untreated cells, are shown. (I) The ubiquitination status of F-MORs or F-MOR K94,96Rs was assessed after treated with 10 μM DADLE for the indicated times before extraction and immunoprecipitation with FLAG antibody. A representative immunoblot detecting endogenous ubiquitin in untransfected cells (293) and in cells expressing F-MOR and F-MOR K94,96R (left) is shown. Blots were probed with antibody against endogenous ubiquitin (middle) or FLAG (right) to control for equal loading and receptor expression. (J) Immunoblots from multiple experiments were scanned to estimate the amount of ubiquitin at each time point after treatment with DADLE and expressed as a fold increase over signal in untransfected 293 cells. The results were pooled and averaged across multiple experiments; n = 4. (K and L) The ubiquitination status of MOR0cK R94,96Ks or MOR0cKs was measured as in (I) and (J); n = 3. Error bars correspond to SEM and p values: one- or two-way ANOVA, Bonferroni multiple comparison test; ^∗^p < 0.05; ^∗∗^p < 0.01; ^∗∗∗^p < 0.001. See also Figure S1 and Movie S2.

To determine if this effect is dependent on a specific site of MOR ubiquitination, we reverted lysine residues in individual cytoplasmic domains in the MOR0cK background (Figure 3A). Restoring only two lysine residues present in the first intracellular loop (MOR0cK R94,96K) fully rescued the endocytic defect, while reverting lysines in any other cytoplasmic domain did not (Figure 3B). Conversely, specifically preventing ubiquitination in the first cytoplasmic loop of MOR (MOR K94,96R) inhibited agonist-induced endocytosis (Figure 3C; Figure S2F). Mutating either of the two first-loop lysine residues individually produced an intermediate phenotype (not shown), prompting us to focus on dual lysine mutation for subsequent analysis.

To determine if first-loop lysine residues affect individual CCP dynamics, we compared mutant MORs containing or lacking only these residues (MOR0cK R94,96K or MOR K94,96R, respectively). Both clustered robustly in response to DADLE, and with indistinguishable clustering times (Figure S1B). However, CCPs containing MOR K94,96Rs exhibited markedly longer surface lifetime, often persisting in the plasma membrane for several minutes before endocytic scission (Figures 3D–3G). Thus, lysine residues in the MOR first cytoplasmic loop, while not required for the formation of CCPs or robust receptor clustering within them, specifically control the surface lifetime of receptor-containing CCPs before they disappear from the plasma membrane.

Ubiquitination of the same cytoplasmic lysine residues promotes topological sorting of MORs from the limiting membrane to intralumenal vesicles of late endosomes/multivesicular bodies (Hislop et al., 2011). Radioligand binding assay verified that this proteolytic effect occurs clearly after endocytosis (Figure 3H), and we established biochemically that first-loop cytoplasmic lysine residues are major sites of agonist-induced MOR ubiquitination (Figures 3I–3L). Together these results indicate that MORs undergo agonist-induced ubiquitination in the first cytoplasmic loop, affecting discrete events of receptor endocytosis and postendocytic sorting.

### MOR Ubiquitination Locally Controls the Maturation of Receptor-Containing CCPs

**Figure 4:**
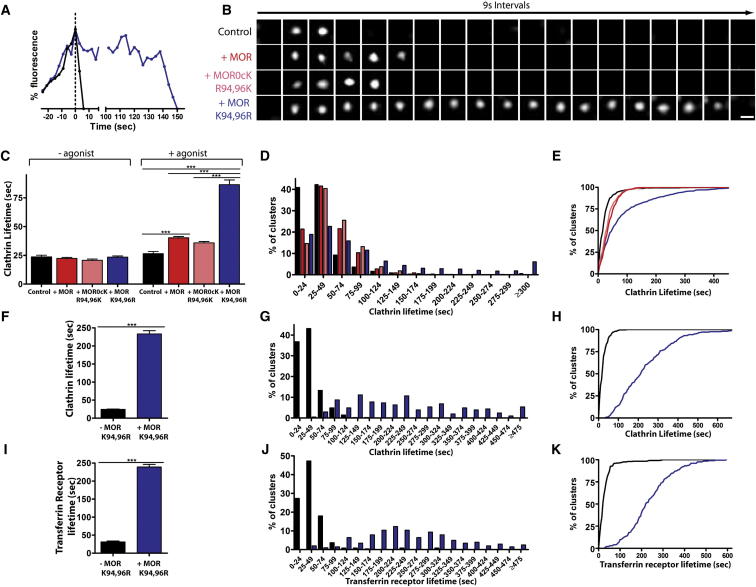
MOR Ubiquitination Decreases the Surface Lifetime of Clathrin and Affects Other Cargo in MOR-Containing CCPs (A) Clathrin fluorescence traces of representative short (black) and long-lived (blue) CCPs in a MOR K94,96R-expressing cell imaged in the presence of agonist. Traces were aligned (t = 0 denotes the end of the ascending phase). (B) Representative image sequences of CCPs from Control, + MOR, +MOR0cK R94,96K, or +MOR K94,96R cells treated with agonist while imaging using TIRF-M. Frames are 9 s apart; scale bar = 500 nm. (C) The average clathrin lifetimes before and after agonist treatment in cells expressing DsRed-tagged clathrin light chain and pCDNA 3.0 (Control), MOR, MOR0cK R94,96K, or MOR K94,96R. Before agonist treatment: Control (black) n = 207 clusters, five cells; +MOR (red) n = 308 clusters, five cells; +MOR0cK R94,96K (pink) n = 135 clusters, five cells, +MOR K94,96R (blue) n = 302 clusters, five cells. After agonist treatment: Control n = 550 clusters, five cells; +MOR n = 607 clusters, five cells; +MOR0cK R94,96K n = 542 clusters, five cells, +MOR K94,96R n = 592 clusters, five cells. (D and E) Frequency distribution (D) and cumulative probability (E) analysis of clathrin surface lifetimes. (F) The average clathrin lifetimes in MOR K94,96R-expressing cells treated with agonist, separated into populations based on whether they lack (−MOR K94,96R) or contain (+MOR K94,96R) receptors measured within the same five cells. −MOR K94,96R n = 413 clusters; +MOR K94,96R n = 206 clusters. (G and H) Frequency distribution (G) and cumulative probability (H) analysis of clathrin cluster lifetimes that either lack (−MOR K94,96R, black) or contain (+MOR K94,96R, blue) MOR K94,96R receptors. (I) The average SpH-Transferrin receptor cluster lifetimes in MOR K94,96R-expressing cells treated with DADLE, separated by whether they lack (−MOR K94,96R) or contain (+MOR K94,96R) mutant receptors. −MOR K94,96R n = 245 clusters, five cells; +MOR K94,96R n = 203 clusters, five cells. (J and K) Frequency distribution (J) and cumulative probability (K) analysis of the lifetimes of transferrin receptor clusters that either lack (−MOR K94,96R, black) or contain (+MOR K94,96R, blue) MOR K94,96R receptors. Error bars indicate standard error of the mean (SEM). p values: one-way ANOVA, Bonferroni multiple comparison test; ^∗∗∗^p < 0.001. See also Figure S3.

To examine the kinetics of CCPs themselves, we next imaged the formation and disappearance of diffraction-limited spots labeled by DsRed-tagged clathrin light chain (Merrifield et al., 2002). In cells not expressing opioid receptors, or in receptor-expressing cells not exposed to opioid agonist, most CCPs labeled in this manner increased in fluorescence intensity over a period of ∼30 s after their initial appearance and then abruptly disappeared from the evanescent field within an additional 5–10 s (Figure 4A, black line shows a representative intensity trace). This behavior is consistent with the previously described dynamics of CCPs at 37°C under conditions of near-endogenous clathrin expression (Doyon et al., 2011; Ehrlich et al., 2004; Kaksonen et al., 2005; Merrifield et al., 2002; Taylor et al., 2011). In cells expressing wild-type MORs and exposed to agonist, we saw the emergence of a subset of clathrin spots with a moderately longer surface lifetime. Mutating lysine residues present in the first cytoplasmic loop exaggerated this delay. Examining individual examples indicated that the delay occurred after clathrin intensity reached a near-maximum value (Figure 4A, blue line shows a representative intensity trace), suggesting that kinetic control is exerted by receptors after clathrin lattice assembly is complete.

Representative TIR-FM imaging series of the range of behaviors observed, from cells expressing each mutant receptor construct examined, are shown in Figure 4B. We verified these observations quantitatively across multiple examples and experiments by determining a mean CCP surface lifetime (Figure 4C). Frequency distribution and cumulative probability plots also revealed a pronounced rightward shift in the distribution of clathrin surface lifetimes that was dependent both on the receptor construct expressed and the presence of agonist (Figures 4D and 4E). A similar effect was evident when the same image series was analyzed using a previously described (Jaqaman et al., 2008) computer algorithm (Figures S3A and S3B).

Because various 7TMRs cluster nonuniformly within the CCP population (Cao et al., 1998; Mundell et al., 2006; Puthenveedu and von Zastrow, 2006), we next asked if the subset of CCPs with prolonged surface lifetime corresponded to those containing activated MORs. To do so, we used dual-label TIR-FM to analyze the CCP lifetime of all diffraction-limited clathrin spots (representing the overall population of CCPs) and then separated the individual determinations according to the presence or absence of MOR K94,96R fluorescence. Increased mean surface lifetime was observed only for the receptor-containing subset (Figure 4F), and the rightward shift in the distribution of surface lifetimes fully segregated with this subset (Figures 4G and 4H). These results suggest that activated opioid receptors locally control CCPs and do so in a manner determined by the receptor’s specific ubiquitination status. In essence, nonubiquitinated MORs act as a “brake” to stall receptor-containing CCPs, while ubiquitination of the receptor’s first cytoplasmic loop functions as a “brake release” to trigger subsequent endocytic scission.

This interpretation predicts that the surface lifetime of another cargo, copackaged with ubiquitin-defective opioid receptors, would also be affected. To test this, we carried out dual-label TIR-FM imaging of mutant opioid receptors together with SpH-tagged transferrin receptors (SpH-TfRs) that cluster constitutively in CCPs. While many SpH-TfR spots exhibited relatively short surface lifetimes, a subset persisted in the plasma membrane for an extended time period before abruptly disappearing from the evanescent field; precisely these spots colocalized with F-MOR K94,96R (Figures 4I–4K).

### Ubiquitination in the First Cytoplasmic Loop Controls MOR Loading into CCPs

**Figure 5:**
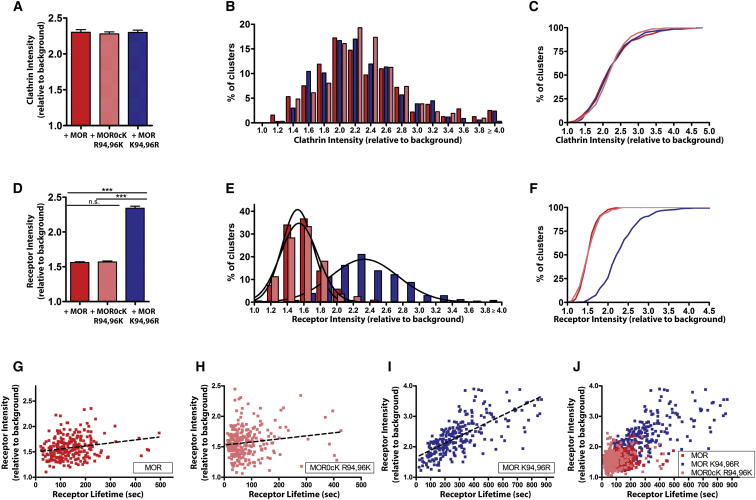
MOR Ubiquitination Controls the Amount of Receptor Loaded into Individual CCPs. (A) The average, normalized clathrin fluorescence intensities in cells expressing clathrin light chain and MOR, MOR0cK R94,96K, or MOR K94,96R were measured, shown as a fold increase in fluorescence over background. MOR n = 319 clusters, seven cells; MOR0cK R94,96K n = 311 clusters, five cells; and MOR K94,96R n = 335 clusters, seven cells. (B and C) Frequency distribution (B) and cumulative probability curves (C) of clathrin intensities in MOR (red), MOR0cK R94,96K-expressing (pink), and MOR K94,96R-expressing (blue) cells. (D) The average, normalized receptor cluster intensities in cells expressing MOR, MOR0cK R94,96K, or MOR K94,96R treated with agonist. MOR n = 301 clusters, seven cells; MOR0cK R94,96K n = 263 clusters, eight cells; and MOR K94,96R n = 292 clusters, seven cells. (E) Frequency distribution of receptor clusters with the specified intensities (bars) was fitted to a Gaussian curve (lines). For MOR, R^2^ = 0.9890, d.f. = 16, Sy.x = 1.257, and coefficient of variation (CV) = 0.131; for MOR0cK R94,96K R^2^ = 0.9958, d.f. = 16, Sy.x = 0.7041, and CV = 0.155, for MOR K94,96R, R^2^ = 0.9535, d.f. = 16, Sy.x = 1.501 and CV = 0.215. (F) Cumulative probability curves for intensity measurements of wild-type or mutant receptors. (G–I) Receptor intensities were plotted against lifetimes for individual clusters to assess any correlation between intensity and receptor lifetime for MOR (G), MOR0cK R94,96K (H), and MOR K94,96R (I). MOR correlation coefficient = 0.2051 and R^2^ = 0.04207; MOR0cK R94,96K correlation coefficient = 0.1486 and R^2^ = 0.02209; MOR K94,96R correlation coefficient = 0.6885 and R^2^ = 0.4740. (J) The compiled results are shown at the same scale. Error bars correspond to SEM and p values: one-way ANOVA, Bonferroni multiple comparison test; ^∗∗∗^p < 0.001.

First-loop mutations did not detectably affect the peak value of clathrin light chain fluorescence measured before CCP scission visualized by TIR-FM (Figures 5A–5C). However, a pronounced difference was observed when the same approach was used to quantify the fluorescence of receptors. Preventing MOR ubiquitination in the first loop increased mean receptor loading of CCPs estimated at the time of scission (Figure 5D), and produced an obvious right shift in the distribution of individual receptor intensity values (Figures 5E and 5F). We noted a detectable but smaller (30%–50%) increase in the coefficient of variation of the intensity distribution (legend to Figure 5E), suggesting that preventing MOR ubiquitination also increases variability in the degree of receptor loading into individual CCPs.

These observations were further supported by detailed analysis of the relationship between ubiquitin-dependent control of CCP surface lifetime and cargo loading. When ubiquitination of the first cytoplasmic loop was allowed to occur (i.e., wild-type MOR or MOR0cK R94,96K), CCP lifetimes were uniformly short and cargo loading (receptor fluorescence intensity measured at individual CCPs) was relatively tightly clustered (Figures 5G and 5H). However, when ubiquitination was prevented (MOR K94,96R), there was an extended distribution of clathrin lifetimes that positively correlated with cargo load (Figures 5I and 5J, correlation coefficient = 0.69). This suggests that by controlling the surface lifetime of individual CCPs, ubiquitination of MOR’s first cytoplasmic loop effectively limits the amount of receptor cargo accumulated into CCPs by the time of endocytic scission.

### Ubiquitin Is Added to Receptors by Activation and Phosphorylation-Dependent Recruitment of Smurf2

**Figure 6:**
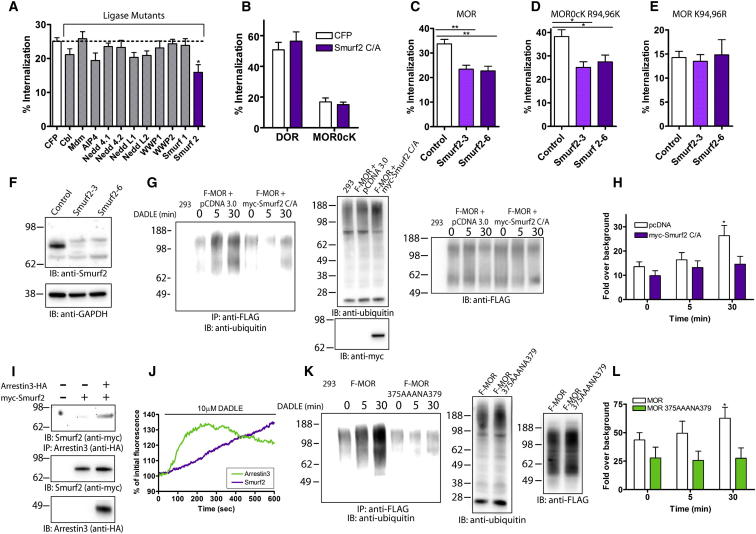
Mechanism of MOR Ubiquitination (A) Flow cytometric analysis of MOR internalization in cells expressing the specified catalytically inactive E3 ligases after 30 min DADLE treatment; n ≥ 3. (B) Flow cytometric analysis of DOR or MOR0cK internalization after 30 min DADLE treatment in cells transfected with CFP or an inactive mutant Smurf2; n = 3. (C–E) The percentage of MOR (C, n = 4), MOR0cK R94,96K (D, n = 6), or MOR K94,96R (E, n = 4) internalization after 30 min DADLE addition in cells transfected with control siRNA or two siRNA duplexes against Smurf2. (F) Representative immunoblot analysis of siRNA-mediated knockdown of Smurf2 with GAPDH loading control; n = 3. (G) The ubiquitination status of MORs was measured in cells transfected with pCDNA 3.0 or myc-Smurf2 C/A after incubation with DADLE for 5 or 30 min. Shown is a representative blot detecting endogenous ubiquitin in untransfected cells (293) and in F-MOR cells expressing pCDNA 3.0 or myc-Smurf2 C/A (left). Loading and expression was assessed by probing for endogenous ubiquitin (top right), FLAG (middle right), and myc (bottom right). (H) Densitometry of immunoblots from multiple experiments was performed and results were averaged across multiple experiments; n = 5. (I) Co-IP of Smurf2 and Arrestin3. Untransfected cells and cells expressing myc-Smurf2 with pCDNA 3.0 or HA-Arrestin3 were extracted and immunoprecipitated with HA antibody before immunoblotting for myc. Blots were probed with antibodies against myc and HA to confirm expression and equal loading. (J) The increase in intensity of Arrestin3 or Smurf2 was measured after agonist addition in F-MOR expressing cells by TIRF-M and is expressed as the percentage of initial fluorescence; n = 12 cells. (K and L) The ubiquitination status of MOR or F-MOR 375AAANA379 receptors was measured as in (G) and (H). A representative blot is shown detecting ubiquitin in untransfected cells and in F-MOR or F-MOR 375AAANA379 cells (left). Equal loading and expression were confirmed by probing lysates for endogenous ubiquitin (middle), and FLAG antibody (right). Densitometry of immunoblots was performed and averaged across multiple experiments; n = 4. p values: two-way ANOVA, Bonferroni multiple comparison test; ^∗^p < 0.05; ^∗∗^p < 0.01. See also Figure S4.

Having established first-loop ubiquitination as the critical biochemical information conferring local control of CCP lifetime, we next investigated the mechanism responsible for adding this signal. We started by identifying the relevant ubiquitin ligase. To do so, we screened for effects of catalytic inactivation, focusing on HECT domain ligases related to Rsp5/Nedd4 because of their widespread endocytic functions and on a subset of RING domain ligases shown previously to function in the endocytic pathway (Hislop and von Zastrow, 2011; Staub and Rotin, 2006). The HECT domain ubiquitin ligase Smurf2 emerged as a strong candidate (Figure 6A), which we pursued further because its effects were highly cargo specific and evident using both mutational inactivation (Figure 6B) and small interfering RNA (siRNA)-mediated knockdown (Figures 6C and 6F). Further, Smurf2 knockdown inhibited internalization of MORs only when lysine residues were present in the first loop, whereas it produced no (additional) inhibitory effect on MORs lacking lysine residues in the first loop (Figures 6D and 6E). Finally, we verified that specifically disrupting Smurf2 catalytic activity inhibited agonist-induced ubiquitination of MORs (Figures 6G and 6H). These observations provide independent lines of genetic and biochemical evidence that Smurf2 regulates MOR endocytosis by adding ubiquitin to the first cytoplasmic loop.

**Figure 7:**
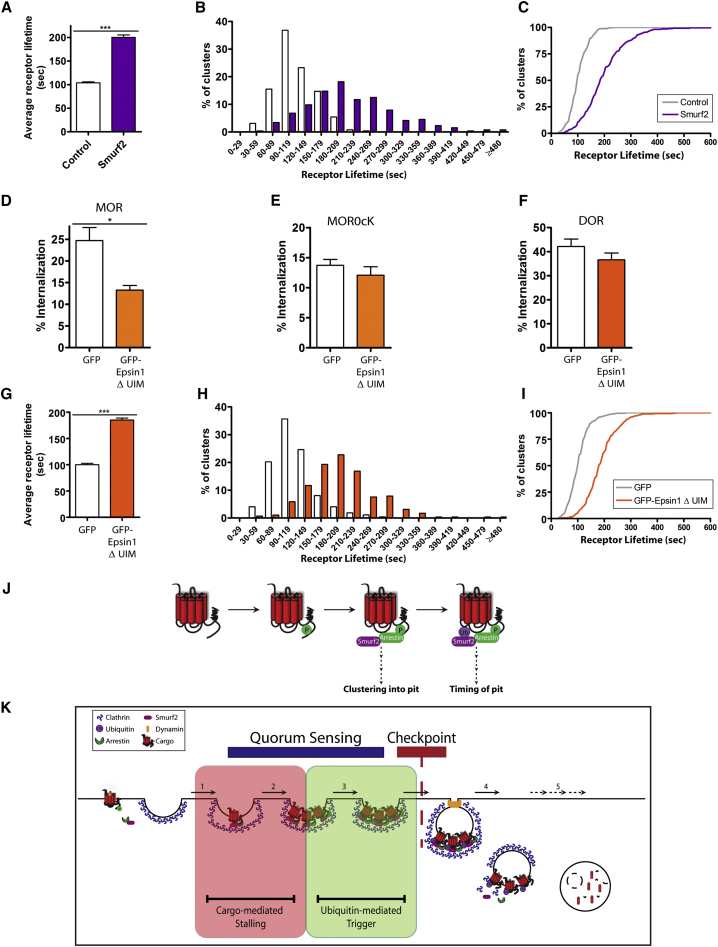
Smurf-2 Mediated Ubiquitination of MOR Controls Receptor Surface Lifetime (A) The average lifetimes of MOR clusters were measured in control or Smurf2 siRNA-expressing cells after agonist treatment; Control n = 258 clusters, nine cells; Smurf2 n = 264 clusters, 11 cells. (B and C) Frequency distribution (B) and cumulative probability (C) analysis of MOR cluster lifetimes in control (white) or Smurf2 (purple) siRNA-transfected cells. (D–F) MOR (D, n = 5), MOR0cK (E, n = 4), or DOR (F, n = 4) internalization after 30 min DADLE was measured in cells expressing receptors and GFP or GFP-Epsin1ΔUIM. (G) The average lifetime measurements of MOR clusters in GFP or GFP-Epsin1ΔUIM-expressing cells after agonist treatment; GFP n = 272 clusters, nine cells; GFP-Epsin1ΔUIM n = 290 clusters, nine cells. (H and I) Frequency distribution (H) and cumulative probability (I) analysis of MOR surface lifetimes in GFP- (white) or GFP-Epsin1ΔUIM-expressing (orange) cells. Error bars correspond to SEM and p values: Student’s t test or one-way ANOVA, Bonferroni multiple comparison test; ^∗^p < 0.05; ^∗∗∗^p < 0.001. (J) Model for the role of posttranslational modifications in MOR endocytosis. After agonist addition, receptors undergo phosphorylation that recruits Arrestin3 and Smurf2 to activated receptors and promotes entry of receptors into CCPs. Smurf2 ubiquitinates MORs, modulating the lifetime of receptor-containing CCPs. (K) Model for the role of receptor-mediated control of CCPs. Receptors are recruited to CCPs via interactions with Arrestin3 (1), where they effectively stall the CCP (2) until receptors undergo Smurf2-mediated ubiquitination (3). This prompts endocytic scission (4) and functions as both a quorum sensor for cargo load and as a checkpoint for later receptor destruction (5). See also Figures S1 and S5.

We next asked how Smurf2-mediated ubiquitination of MORs is coordinated with agonist-induced receptor activation and clustering. Smurf2 associated with Arrestin3 (beta-arrestin2) in cell extracts (Figure 6I), and Arrestin3 is already known to undergo agonist-induced recruitment to MORs (Cen et al., 2001; Johnson et al., 2006; Zhang et al., 1998). This suggested that Arrestin3, in addition to functioning as an endocytic adaptor for MORs, might also function as a scaffold for recruiting Smurf2 to agonist-activated MORs. Supporting this hypothesis, both Arrestin3-GFP and Smurf2-GFP fluorescence rapidly increased at the plasma membrane after receptor activation, with Smurf2 recruitment following that of Arrestin3 (Figure 6J, green and purple lines, respectively). Further, Smurf2 accumulated both diffusely and in spots in the plasma membrane (Figure S4) reminiscent of Arrestin3 localization shown previously (Puthenveedu and von Zastrow, 2006), and recruitment of both proteins began before detectable receptor clustering in CCPs (compare Figure 6J with Figures S1A and S1C). Beta-arrestins associate with MORs following agonist-induced phosphorylation of the receptor’s cytoplasmic tail (Groer et al., 2011; Johnson et al., 2006; Zhang et al., 1998), and the specific phosphorylation sites required for this recruitment were recently defined (Lau et al., 2011). Mutating only these residues (F-MOR 375AAANA379) blocked agonist-induced ubiquitination of MORs (Figures 6K and 6L). Further, knockdown of Smurf2 effectively phenocopied lysyl-mutation of MORs, as indicated by a significant increase in mean receptor surface lifetime (Figure 7A) and a pronounced right shift in the frequency distribution (Figures 7B and 7C). Additionally, expression of GFP-tagged, catalytically inactive mutant Smurf2 resulted in ∼2-fold increase in MOR surface lifetime compared to expression of GFP alone (data not shown), similar to the effect of Smurf2 knockdown. These results suggest that Smurf2-mediated ubiquitination of MORs is controlled, and coordinated with receptor clustering in CCPs, through arrestin-mediated recruitment.

### The Ubiquitin Signal Is Detected at CCPs by Epsin1

To determine how the ubiquitin-encoded signal is detected to transduce local CCP control, we screened CCP-associated proteins that are known to bind ubiquitin. Obvious candidates are epsins, a conserved family of ubiquitin-interacting motif (UIM)-containing proteins that assemble with CCPs (Chen et al., 1998; Kazazic et al., 2009; Shih et al., 2002; Wendland, 2002), and whose knockdown or overexpression can alter CCP dynamics (Mettlen et al., 2009) or endocytic function (Chen et al., 2011; Rosenthal et al., 1999; Sorkina et al., 2006; Sugiyama et al., 2005). siRNA duplexes targeting Epsin2 did not detectably affect MOR internalization (not shown) but Epsin1 depletion caused a significant inhibition (Figure S5A). We initially rejected this candidate because its knockdown (Figures S5B and S5C) and overexpression (Figures S5D and S5E) also affected lysyl mutant MORs, consistent with these manipulations of overall Epsin1 abundance causing a general impairment of endocytosis. We later reconsidered this candidate because deleting only the tandem UIMs (Epsin1ΔUIM), which disrupts Epsin1 binding to ubiquitinated proteins (Sugiyama et al., 2005), did not detectably change Epsin1 distribution or assembly with CCPs (Figures S5F and S5G) and affected MOR endocytosis in a ubiquitination-specific manner: Epsin1ΔUIM inhibited internalization of the wild-type MOR (Figure 7D) but had no detectable effect on internalization of the lysyl mutant MOR0cK, which slows CCP maturation but is unable to undergo the critical first-loop ubiquitination (Figure 7E), or of the DOR that does not delay CCP maturation in the first place (Figure 7F). Further supporting this conclusion, Epsin1ΔUIM prolonged surface lifetime of CCPs containing wild-type MORs (Figure 7G–7I) without impairing receptor clustering in CCPs initiated by agonist-induced receptor activation (Figure S1E). These results experimentally isolate a specific function of Epsin1, through its UIMs, in recognizing appropriately ubiquitinated MORs and triggering endocytic scission of the CCPs containing them.

## Discussion

The present results show that a signaling receptor that is itself regulated by CCP-mediated endocytosis can, conversely, exert active and local cargo-dependent control over the endocytic process. We observed that MORs prolong the surface lifetime of receptor-containing CCPs after their formation and initial cargo accumulation, but before the occurrence of dynamin-dependent scission, verifying and extending the observation that some 7TMRs exert a “brake” function on the maturation of their enclosing CCPs (Puthenveedu and von Zastrow, 2006). Ubiquitination of MORs in the first cytoplasmic loop conveys an active biochemical signal that effectively counteracts this inhibitory effect, allowing specific receptor ubiquitination to act as an endocytic “brake release.” The present results add to the accumulating evidence supporting cargo-mediated regulation at various stages in the conserved CCP pathway (Ehrlich et al., 2004; Liu et al., 2010; Loerke et al., 2009; Mettlen et al., 2010; Puthenveedu and von Zastrow, 2006; Santini et al., 2002), and reveal an additional level of control through local signaling based on cargo ubiquitination.

Ubiquitination of endocytic cargoes is well known to promote the initial accumulation in CCPs through ubiquitin-binding endocytic adaptors (Hicke and Dunn, 2003; Maldonado-Báez and Wendland, 2006; Shih et al., 2002; Torrisi et al., 1999; Toshima et al., 2009), and to promote later multivesicular body/lysosome sorting of endocytic cargo through interactions with the ESCRT machinery (Raiborg and Stenmark, 2009; Saksena et al., 2007; Shields and Piper, 2011). The present results identify a discrete function of ubiquitin as a biochemical signal that actively controls CCP maturation. Our results add to accumulating evidence for additional endocytic functions of ubiquitin (Jiang and Sorkin, 2003; Reider and Wendland, 2011) and, taken in context with recent evidence for ubiquitin-dependent control in the biosynthetic pathway (Jin et al., 2012), suggest a potentially widespread role of ubiquitin as a cargo-specific regulator of coat protein dynamics.

Ubiquitin is added to the receptor by Arrestin3 -dependent recruitment of the Smurf2 ubiquitin ligase, promoted by specific phosphorylation of the MOR cytoplasmic tail. This mechanism for regulating receptor ubiquitination intrinsically assures close coordination of ubiquitination with receptor activation and clustering into CCPs (Figure 7J). We are not aware of previous evidence for such a biochemical encoding mechanism for opioid receptors, or for Smurf2 specifically, but regulation by arrestin-mediated recruitment of ubiquitin ligases is emerging as a repeated theme in cell biology (Bhandari et al., 2007; Shenoy et al., 2001, 2008). The regulatory effect of receptor ubiquitination on CCP surface lifetime requires epsin1, and specifically its UIMs, identifying a discrete function of epsin in sensing and transducing the endocytosis-promoting activity of this local biochemical signal.

Ubiquitin-dependent endocytic control appears to mediate a biochemical “checkpoint” that delays endocytosis until appropriate ubiquitination (Hislop et al., 2011) of MORs is achieved to facilitate later trafficking (Figure 7F, red bar). We also found that first-loop ubiquitination affects MOR density in individual CCPs at the time of endocytic scission (Figures 5D–5J), suggesting that it represents a simple biochemical mechanism to estimate local concentration of a specific endocytic cargo and control CCP surface lifetime accordingly (Figure 7K, red and green boxes). The latter effect is reminiscent of “quorum sensing” (Miller and Bassler, 2001) described initially in bacterial ecology (Fuqua et al., 1994; Nealson et al., 1970), except that the presently described density-measurement system is based on local accumulation of covalently attached ubiquitin rather than of a secreted factor (Fuqua and Greenberg, 1998; Hastings and Greenberg, 1999; Jayaraman and Wood, 2008). Thus, we propose that the presently identified cargo-to-coat communication system functions, at the systems level, as a means to fundamentally and locally tailor the endocytic pathway to the needs of specialized membrane cargoes such as signaling receptors.

## Experimental Procedures

### Expression Constructs, Reagents, and Statistical Analysis

Expression constructs and reagents, as well as statistical methods, are described in Supplemental Experimental Procedures. Other methods are summarized below, with additional detail included in Supplemental Experimental Procedures.

### Flow Cytometric Analysis of Endocytosis

The flow cytometric assay was carried out as previously described (Hislop et al., 2011; Tsao and von Zastrow, 2000).

### TIR-FM Imaging

TIR-FM was performed at 37°C using a Nikon TE-2000E inverted microscope with a 60× 1.45 NA TIRF objective, using through-the-objective illumination and 488 nm Argon-ion laser (Melles Griot) and 543 nm HeNe laser (Spectra Physics) as light sources. An iXon (Andor) camera was used to acquire image sequences, controlled by Andor IQ software.

### Immunoelectron Microscopy of Plasma Membrane Sheets

Cell surface receptor was detected by sequential incubation with 10 μg/ml rabbit anti-FLAG IgG (Sigma-Aldrich, MO, USA) and protein A conjugated to 10 nm gold particles, after which the cells were washed in HEPES buffer (25 mM HEPES, 25 mM KCl, and 2.5 mM Mg acetate [pH 7.0]). Plasma membrane sheets were prepared using a previously described “rip-off” technique (Sanan and Anderson, 1991; Signoret et al., 2005). Images were acquired using a Tecnai G2 Spirit transmission EM (FEI, Eindhoven, the Netherlands) fitted with a Morada 11 MegaPixel TEM camera (Olympus Soft Imaging Solutions, Münster, Germany).

### Image Analysis

Image analysis was performed using ImageJ software (Wayne Rasband, NIH). For surface lifetime measurements, the time between the appearance and disappearance of clusters was measured as previously described (Puthenveedu and von Zastrow, 2006). For cluster intensity measurements, the mean intensity value of clusters immediately prior to endocytic scission and of an identically sized background region of the cell at that same frame was quantified. Fluorescence values of clusters were divided by those of background regions and expressed as “Fold over background” values. For change in intensity over time measurements, regions of interest were drawn around individual cells and the change in integrated intensity was measured and background-corrected. These values were normalized to the intensity of the cell immediately prior to agonist treatment and are represented as “% of initial fluorescence” values.

### Analysis of Receptor Degradation by Radioligand Binding

The amount of receptor remaining after prolonged agonist treatment was measured by radioligand binding, as previously described (Hislop et al., 2009; Tsao and von Zastrow, 2000). Specific binding values are shown, measured as total binding minus the nonspecific binding at each time point and expressed as the percentage of the binding in untreated cells.

### Detection of Ubiquitination by Western Blot Analysis

Cells stably expressing FLAG-tagged receptors were treated with agonist for the indicated time points and prepared for western blot analysis to detect receptor ubiquitination as previously described (Hislop et al., 2011). Densitometric analysis of band intensities from unsaturated immunoblots were analyzed and quantified by densitometry using FluorChem 2.0 software (Alpha Innotech).

### Coimmunoprecipitation

For immunoprecipitation studies, cells were transiently transfected, lysed in IP buffer supplemented with standard protease inhibitor cocktail (Roche Applied Science), and clarified by centrifugation. Samples were incubated with anti-myc, clone 9E10 antibody (Millipore) overnight and later with protein A/G beads (Pierce), washed, and analyzed by western blotting. Immunoblots were probed with anti-HA antibody (Covance).

## Supplemental Information

Document S1. Figures S1–S5 and Supplemental Experimental Procedures

Movie S1. Both MOR and MOR0cK Cluster into CCPs after Agonist Addition, Related to Figure 2 Cells expressing DsRed-tagged clathrin light chain (shown in red) and either F-MOR or F-MOR0cK (shown in green) labeled with anti-Flag antibody conjugated to Alexa 488 were imaged live using TIR-FM. Shown is a representative movie of cells treated with agonist at t = 0; scale bar, 3 μm.

Movie S2. Mutating Lysine Residues in the First Intracellular Loop of MOR Prolongs the Surface Lifetime of Receptors, Related to Figure 3 Cells expressing either F-MOR0cK R94,96K (left) or F-MOR K94,96R (right) were labeled with anti-Flag antibody conjugated to Alexa 555 and imaged live using TIR-FM. Shown is a representative movie of cells treated with agonist at t = 0; scale bar, 3 μm.

## References

[bib1] Bhandari D., Trejo J., Benovic J.L., Marchese A. (2007). Arrestin-2 interacts with the ubiquitin-protein isopeptide ligase atrophin-interacting protein 4 and mediates endosomal sorting of the chemokine receptor CXCR4. J. Biol. Chem..

[bib2] Brodsky F.M., Chen C.Y., Knuehl C., Towler M.C., Wakeham D.E. (2001). Biological basket weaving: formation and function of clathrin-coated vesicles. Annu. Rev. Cell Dev. Biol..

[bib3] Cao T.T., Mays R.W., von Zastrow M. (1998). Regulated endocytosis of G-protein-coupled receptors by a biochemically and functionally distinct subpopulation of clathrin-coated pits. J. Biol. Chem..

[bib4] Cen B., Xiong Y., Ma L., Pei G. (2001). Direct and differential interaction of beta-arrestins with the intracellular domains of different opioid receptors. Mol. Pharmacol..

[bib5] Chen B., Dores M.R., Grimsey N., Canto I., Barker B.L., Trejo J. (2011). Adaptor protein complex-2 (AP-2) and epsin-1 mediate protease-activated receptor-1 internalization via phosphorylation- and ubiquitination-dependent sorting signals. J. Biol. Chem..

[bib6] Chen H., Fre S., Slepnev V.I., Capua M.R., Takei K., Butler M.H., Di Fiore P.P., De Camilli P. (1998). Epsin is an EH-domain-binding protein implicated in clathrin-mediated endocytosis. Nature.

[bib7] Doyon J.B., Zeitler B., Cheng J., Cheng A.T., Cherone J.M., Santiago Y., Lee A.H., Vo T.D., Doyon Y., Miller J.C. (2011). Rapid and efficient clathrin-mediated endocytosis revealed in genome-edited mammalian cells. Nat. Cell Biol..

[bib8] Ehrlich M., Boll W., Van Oijen A., Hariharan R., Chandran K., Nibert M.L., Kirchhausen T. (2004). Endocytosis by random initiation and stabilization of clathrin-coated pits. Cell.

[bib9] Farsad K., De Camilli P. (2003). Mechanisms of membrane deformation. Curr. Opin. Cell Biol..

[bib10] Fuqua C., Greenberg E.P. (1998). Self perception in bacteria: quorum sensing with acylated homoserine lactones. Curr. Opin. Microbiol..

[bib11] Fuqua W.C., Winans S.C., Greenberg E.P. (1994). Quorum sensing in bacteria: the LuxR-LuxI family of cell density-responsive transcriptional regulators. J. Bacteriol..

[bib12] Groer C.E., Schmid C.L., Jaeger A.M., Bohn L.M. (2011). Agonist-directed interactions with specific beta-arrestins determine mu-opioid receptor trafficking, ubiquitination, and dephosphorylation. J. Biol. Chem..

[bib13] Hastings J.W., Greenberg E.P. (1999). Quorum sensing: the explanation of a curious phenomenon reveals a common characteristic of bacteria. J. Bacteriol..

[bib14] Hicke L., Dunn R. (2003). Regulation of membrane protein transport by ubiquitin and ubiquitin-binding proteins. Annu. Rev. Cell Dev. Biol..

[bib15] Hislop J.N., von Zastrow M. (2011). Role of ubiquitination in endocytic trafficking of G-protein-coupled receptors. Traffic.

[bib16] Hislop J.N., Henry A.G., Marchese A., von Zastrow M. (2009). Ubiquitination regulates proteolytic processing of G protein-coupled receptors after their sorting to lysosomes. J. Biol. Chem..

[bib17] Hislop J.N., Henry A.G., von Zastrow M. (2011). Ubiquitination in the first cytoplasmic loop of μ-opioid receptors reveals a hierarchical mechanism of lysosomal down-regulation. J. Biol. Chem..

[bib18] Itoh T., De Camilli P. (2006). BAR, F-BAR (EFC) and ENTH/ANTH domains in the regulation of membrane-cytosol interfaces and membrane curvature. Biochim. Biophys. Acta.

[bib19] Jaqaman K., Loerke D., Mettlen M., Kuwata H., Grinstein S., Schmid S.L., Danuser G. (2008). Robust single-particle tracking in live-cell time-lapse sequences. Nat. Methods.

[bib20] Jayaraman A., Wood T.K. (2008). Bacterial quorum sensing: signals, circuits, and implications for biofilms and disease. Annu. Rev. Biomed. Eng..

[bib21] Jiang X., Sorkin A. (2003). Epidermal growth factor receptor internalization through clathrin-coated pits requires Cbl RING finger and proline-rich domains but not receptor polyubiquitylation. Traffic.

[bib22] Jin L., Pahuja K.B., Wickliffe K.E., Gorur A., Baumgärtel C., Schekman R., Rape M. (2012). Ubiquitin-dependent regulation of COPII coat size and function. Nature.

[bib23] Johnson E.A., Oldfield S., Braksator E., Gonzalez-Cuello A., Couch D., Hall K.J., Mundell S.J., Bailey C.P., Kelly E., Henderson G. (2006). Agonist-selective mechanisms of mu-opioid receptor desensitization in human embryonic kidney 293 cells. Mol. Pharmacol..

[bib24] Kaksonen M., Toret C.P., Drubin D.G. (2005). A modular design for the clathrin- and actin-mediated endocytosis machinery. Cell.

[bib25] Kasai R.S., Suzuki K.G., Prossnitz E.R., Koyama-Honda I., Nakada C., Fujiwara T.K., Kusumi A. (2011). Full characterization of GPCR monomer-dimer dynamic equilibrium by single molecule imaging. J. Cell Biol..

[bib26] Kazazic M., Bertelsen V., Pedersen K.W., Vuong T.T., Grandal M.V., Rødland M.S., Traub L.M., Stang E., Madshus I.H. (2009). Epsin 1 is involved in recruitment of ubiquitinated EGF receptors into clathrin-coated pits. Traffic.

[bib27] Khelashvili G., Dorff K., Shan J., Camacho-Artacho M., Skrabanek L., Vroling B., Bouvier M., Devi L.A., George S.R., Javitch J.A. (2010). GPCR-OKB: the G Protein Coupled Receptor Oligomer Knowledge Base. Bioinformatics.

[bib28] Kirchhausen T. (2009). Imaging endocytic clathrin structures in living cells. Trends Cell Biol..

[bib29] Lau E.K., Trester-Zedlitz M., Trinidad J.C., Kotowski S.J., Krutchinsky A.N., Burlingame A.L., von Zastrow M. (2011). Quantitative encoding of the effect of a partial agonist on individual opioid receptors by multisite phosphorylation and threshold detection. Sci. Signal..

[bib30] Le Roy C., Wrana J.L. (2005). Clathrin- and non-clathrin-mediated endocytic regulation of cell signalling. Nat. Rev. Mol. Cell Biol..

[bib31] Liu A.P., Aguet F., Danuser G., Schmid S.L. (2010). Local clustering of transferrin receptors promotes clathrin-coated pit initiation. J. Cell Biol..

[bib32] Loerke D., Mettlen M., Yarar D., Jaqaman K., Jaqaman H., Danuser G., Schmid S.L. (2009). Cargo and dynamin regulate clathrin-coated pit maturation. PLoS Biol..

[bib33] Maldonado-Báez L., Wendland B. (2006). Endocytic adaptors: recruiters, coordinators and regulators. Trends Cell Biol..

[bib34] McMahon H.T., Gallop J.L. (2005). Membrane curvature and mechanisms of dynamic cell membrane remodelling. Nature.

[bib35] Merrifield C.J., Feldman M.E., Wan L., Almers W. (2002). Imaging actin and dynamin recruitment during invagination of single clathrin-coated pits. Nat. Cell Biol..

[bib36] Mettlen M., Stoeber M., Loerke D., Antonescu C.N., Danuser G., Schmid S.L. (2009). Endocytic accessory proteins are functionally distinguished by their differential effects on the maturation of clathrin-coated pits. Mol. Biol. Cell.

[bib37] Mettlen M., Loerke D., Yarar D., Danuser G., Schmid S.L. (2010). Cargo- and adaptor-specific mechanisms regulate clathrin-mediated endocytosis. J. Cell Biol..

[bib38] Miesenböck G., De Angelis D.A., Rothman J.E. (1998). Visualizing secretion and synaptic transmission with pH-sensitive green fluorescent proteins. Nature.

[bib39] Miller M.B., Bassler B.L. (2001). Quorum sensing in bacteria. Annu. Rev. Microbiol..

[bib40] Mundell S.J., Luo J., Benovic J.L., Conley P.B., Poole A.W. (2006). Distinct clathrin-coated pits sort different G protein-coupled receptor cargo. Traffic.

[bib41] Nealson K.H., Platt T., Hastings J.W. (1970). Cellular control of the synthesis and activity of the bacterial luminescent system. J. Bacteriol..

[bib42] Perrais D., Merrifield C.J. (2005). Dynamics of endocytic vesicle creation. Dev. Cell.

[bib43] Puthenveedu M.A., von Zastrow M. (2006). Cargo regulates clathrin-coated pit dynamics. Cell.

[bib44] Raiborg C., Stenmark H. (2009). The ESCRT machinery in endosomal sorting of ubiquitylated membrane proteins. Nature.

[bib45] Reider A., Wendland B. (2011). Endocytic adaptors—social networking at the plasma membrane. J. Cell Sci..

[bib46] Rosenthal J.A., Chen H., Slepnev V.I., Pellegrini L., Salcini A.E., Di Fiore P.P., De Camilli P. (1999). The epsins define a family of proteins that interact with components of the clathrin coat and contain a new protein module. J. Biol. Chem..

[bib47] Rust M.J., Lakadamyali M., Zhang F., Zhuang X. (2004). Assembly of endocytic machinery around individual influenza viruses during viral entry. Nat. Struct. Mol. Biol..

[bib48] Saksena S., Sun J., Chu T., Emr S.D. (2007). ESCRTing proteins in the endocytic pathway. Trends Biochem. Sci..

[bib49] Sanan D.A., Anderson R.G. (1991). Simultaneous visualization of LDL receptor distribution and clathrin lattices on membranes torn from the upper surface of cultured cells. J. Histochem. Cytochem..

[bib50] Santini F., Gaidarov I., Keen J.H. (2002). G protein-coupled receptor/arrestin3 modulation of the endocytic machinery. J. Cell Biol..

[bib51] Shenoy S.K., McDonald P.H., Kohout T.A., Lefkowitz R.J. (2001). Regulation of receptor fate by ubiquitination of activated beta 2-adrenergic receptor and beta-arrestin. Science.

[bib52] Shenoy S.K., Xiao K., Venkataramanan V., Snyder P.M., Freedman N.J., Weissman A.M. (2008). Nedd4 mediates agonist-dependent ubiquitination, lysosomal targeting, and degradation of the beta2-adrenergic receptor. J. Biol. Chem..

[bib53] Shields S.B., Piper R.C. (2011). How ubiquitin functions with ESCRTs. Traffic.

[bib54] Shih S.C., Katzmann D.J., Schnell J.D., Sutanto M., Emr S.D., Hicke L. (2002). Epsins and Vps27p/Hrs contain ubiquitin-binding domains that function in receptor endocytosis. Nat. Cell Biol..

[bib55] Signoret N., Hewlett L., Wavre S., Pelchen-Matthews A., Oppermann M., Marsh M. (2005). Agonist-induced endocytosis of CC chemokine receptor 5 is clathrin dependent. Mol. Biol. Cell.

[bib56] Sorkin A., von Zastrow M. (2009). Endocytosis and signalling: intertwining molecular networks. Nat. Rev. Mol. Cell Biol..

[bib57] Sorkina T., Miranda M., Dionne K.R., Hoover B.R., Zahniser N.R., Sorkin A. (2006). RNA interference screen reveals an essential role of Nedd4-2 in dopamine transporter ubiquitination and endocytosis. J. Neurosci..

[bib58] Staub O., Rotin D. (2006). Role of ubiquitylation in cellular membrane transport. Physiol. Rev..

[bib59] Sugiyama S., Kishida S., Chayama K., Koyama S., Kikuchi A. (2005). Ubiquitin-interacting motifs of Epsin are involved in the regulation of insulin-dependent endocytosis. J. Biochem..

[bib60] Taylor M.J., Perrais D., Merrifield C.J. (2011). A high precision survey of the molecular dynamics of mammalian clathrin-mediated endocytosis. PLoS Biol..

[bib61] Taylor M.J., Lampe M., Merrifield C.J. (2012). A feedback loop between dynamin and actin recruitment during clathrin-mediated endocytosis. PLoS Biol..

[bib62] Toret C.P., Drubin D.G. (2006). The budding yeast endocytic pathway. J. Cell Sci..

[bib63] Torrisi M.R., Lotti L.V., Belleudi F., Gradini R., Salcini A.E., Confalonieri S., Pelicci P.G., Di Fiore P.P. (1999). Eps15 is recruited to the plasma membrane upon epidermal growth factor receptor activation and localizes to components of the endocytic pathway during receptor internalization. Mol. Biol. Cell.

[bib64] Toshima J.Y., Nakanishi J.I., Mizuno K., Toshima J., Drubin D.G. (2009). Requirements for recruitment of a G protein-coupled receptor to clathrin-coated pits in budding yeast. Mol. Biol. Cell.

[bib65] Traub L.M. (2009). Tickets to ride: selecting cargo for clathrin-regulated internalization. Nat. Rev. Mol. Cell Biol..

[bib66] Tsao P.I., von Zastrow M. (2000). Type-specific sorting of G protein-coupled receptors after endocytosis. J. Biol. Chem..

[bib67] Weinberg J., Drubin D.G. (2011). Clathrin-mediated endocytosis in budding yeast. Trends Cell Biol..

[bib68] Wendland B. (2002). Epsins: adaptors in endocytosis?. Nat. Rev. Mol. Cell Biol..

[bib69] Zhang J., Ferguson S.S., Barak L.S., Bodduluri S.R., Laporte S.A., Law P.Y., Caron M.G. (1998). Role for G protein-coupled receptor kinase in agonist-specific regulation of mu-opioid receptor responsiveness. Proc. Natl. Acad. Sci. USA.

