# Supplementary figures and images for: Regulation of Endocytic Clathrin Dynamics by Cargo Ubiquitination
Source: Dev Cell. 2012 Sep 11;23(3):519–32. doi: 10.1016/j.devcel.2012.08.003 (PMC3470869; doi:10.1016/j.devcel.2012.08.003)

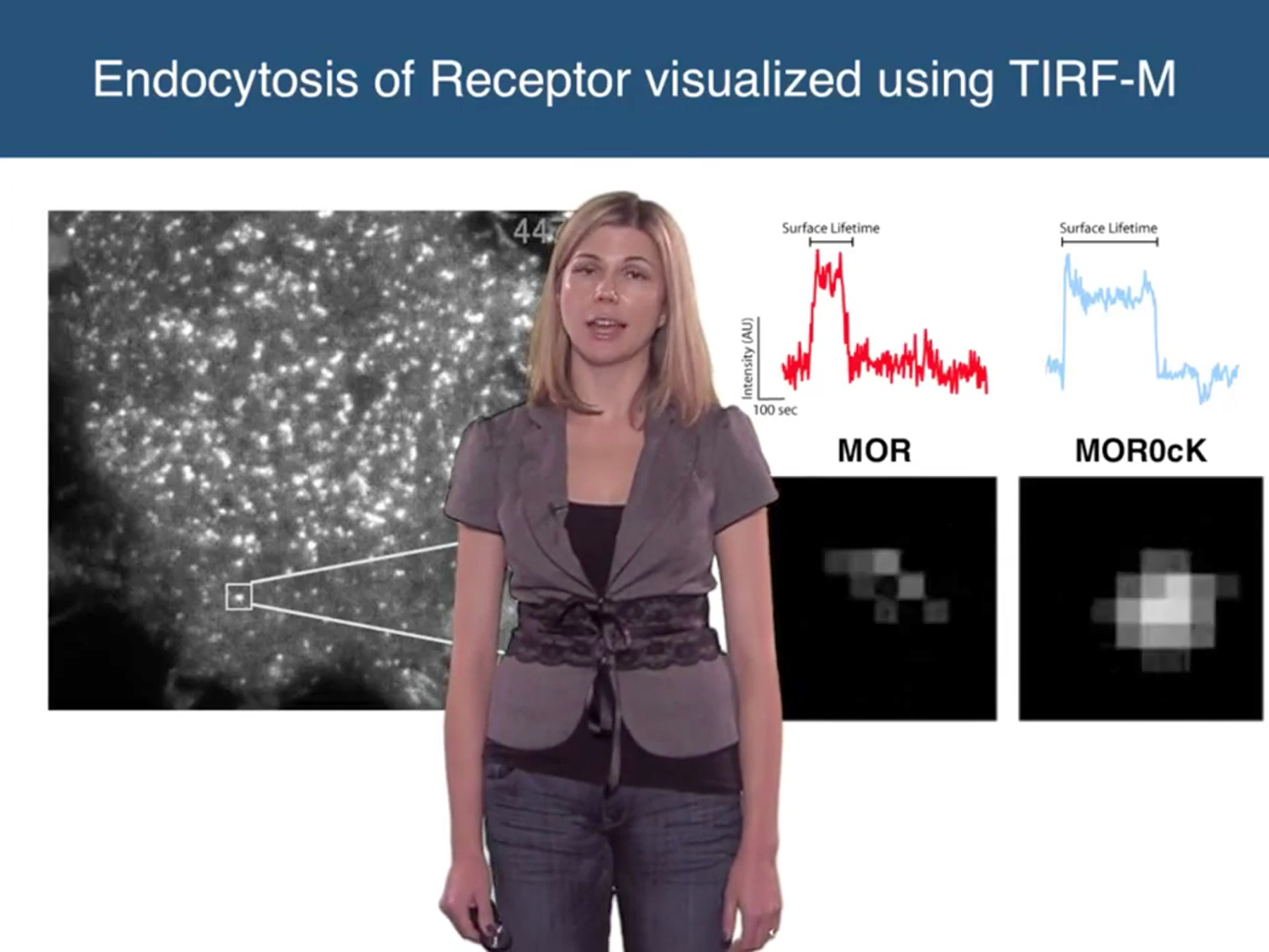

Supplement: Supplementary file 1 [file mmc4.jpg]

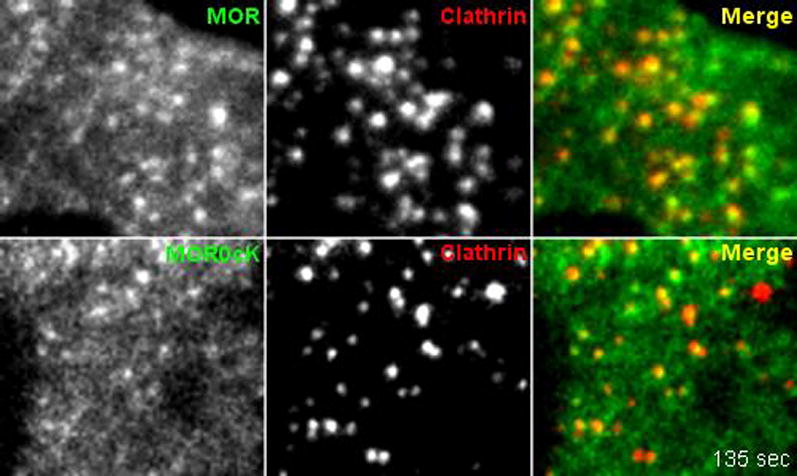

Supplement: Movie S1. Both MOR and MOR0cK Cluster into CCPs after Agonist Addition, Related to Figure 2 — Cells expressing DsRed-tagged clathrin light chain (shown in red) and either F-MOR or F-MOR0cK (shown in green) labeled with anti-Flag antibody conjugated to Alexa 488 were imaged live using TIR-FM. Shown is a representative movie of cells treated with agonist at t = 0; scale bar, 3 μm. [file mmc2.jpg]

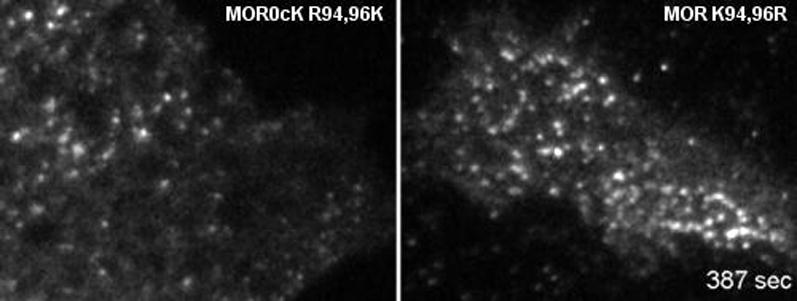

Supplement: Movie S2. Mutating Lysine Residues in the First Intracellular Loop of MOR Prolongs the Surface Lifetime of Receptors, Related to Figure 3 — Cells expressing either F-MOR0cK R94,96K (left) or F-MOR K94,96R (right) were labeled with anti-Flag antibody conjugated to Alexa 555 and imaged live using TIR-FM. Shown is a representative movie of cells treated with agonist at t = 0; scale bar, 3 μm. [file mmc3.jpg]
