# Supplementary material for: Regulation of Endocytic Clathrin Dynamics by Cargo Ubiquitination
Source: Dev Cell. 2012 Sep 11;23(3):519–32. doi: 10.1016/j.devcel.2012.08.003 (PMC3470869; doi:10.1016/j.devcel.2012.08.003)
Supplement: Document S1. Figures S1–S5 and Supplemental Experimental Procedures [file mmc1.pdf]

Developmental Cell, Volume *23*

Supplemental Information

Regulation of Endocytic Clathrin Dynamics by Cargo Ubiquitination

Anastasia G. Henry, James N. Hislop, Joe Grove, Kurt Thorn, Mark Marsh, and Mark von Zastrow

Inventory of Supplemental Information

Supplemental Figures

- Figure S1. Compiled analysis of the time until appearance of receptor-containing CCPs under different conditions that affect receptor ubiquitination, related to Figure 2, 3, and 7.
- Figure S2. The role of receptor ubiquitination on MOR endocytosis measured using SpH-tagged receptors imaged in TIRF-M, related to Figure 2.
- Figure S3. Automated analysis of overall clathrin lifetimes in receptor-expressing cells, related to Figure 4.
- Figure S4. Smurf2 localizes both diffusely and in CCPs after agonist-induced recruitment, related to Figure 6.
- Figure S5. Overexpression or knockdown of Epsin1 inhibits MOR endocytosis irrespective of lysyl-mutation, related to Figure 7.

Supplemental Experimental Procedures

- Expression Constructs and other reagents
- TIR-FM Imaging
- Immunoelectron microscopy of plasma membrane sheets

- Image Analysis
- Detection of ubiquitination by Western blot analysis
- Automated CCP lifetime analysis
- Statistical analysis

Supplemental References

Figure S1

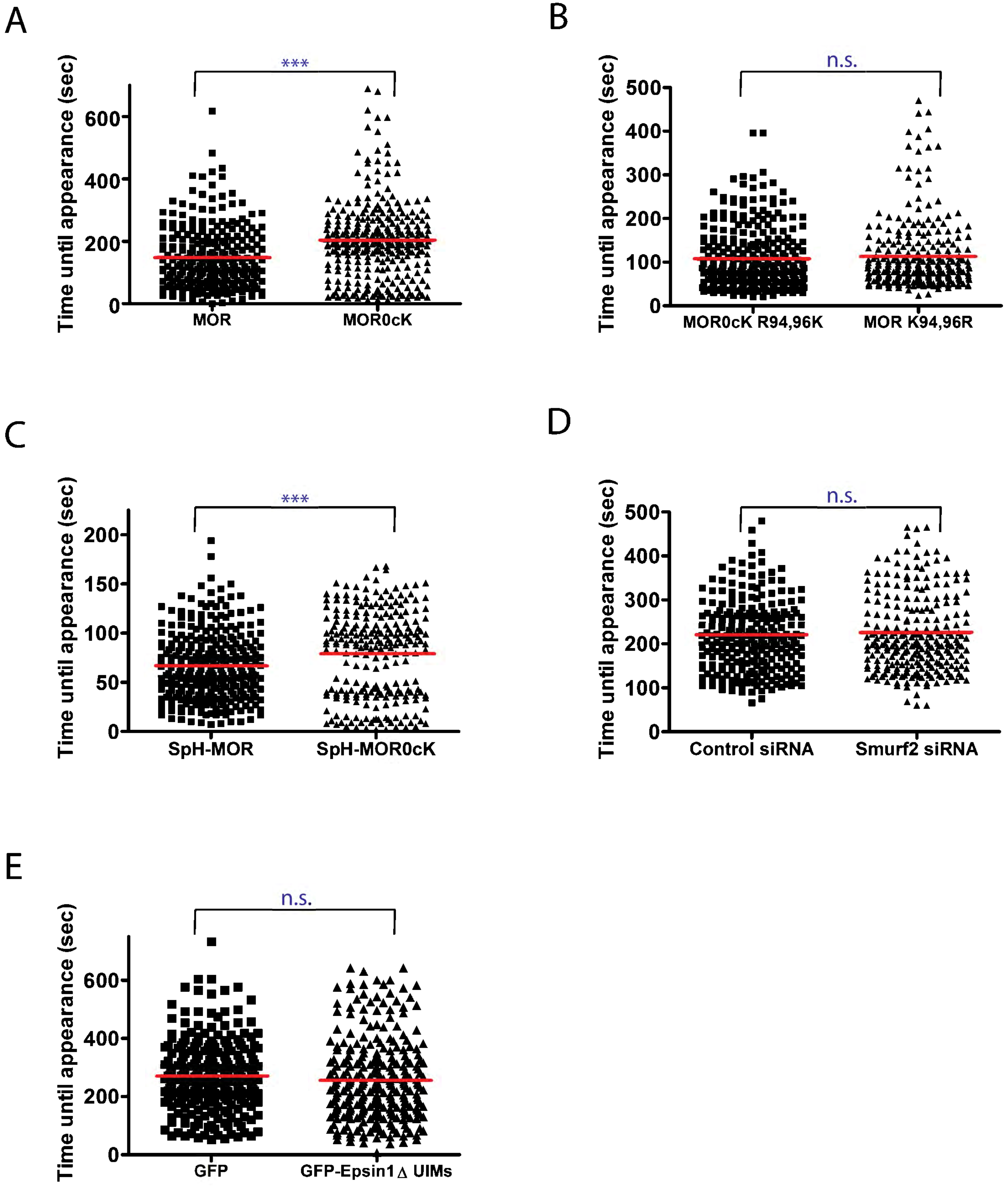
A
***
Time until appearance (sec)
600
400
200
0
MOR
MOR0cK
B
n.s.
Time until appearance (sec)
500
400
300
200
100
0
MOR0cK R94,96K
MOR K94,96R
C
***
Time until appearance (sec)
200
150
100
50
0
SpH-MOR
SpH-MOR0cK
D
n.s.
Time until appearance (sec)
500
400
300
200
100
0
Control siRNA
Smurf2 siRNA
E
n.s.
Time until appearance (sec)
600
400
200
0
GFP
GFP-Epsin1Δ UIMs

Figure S2

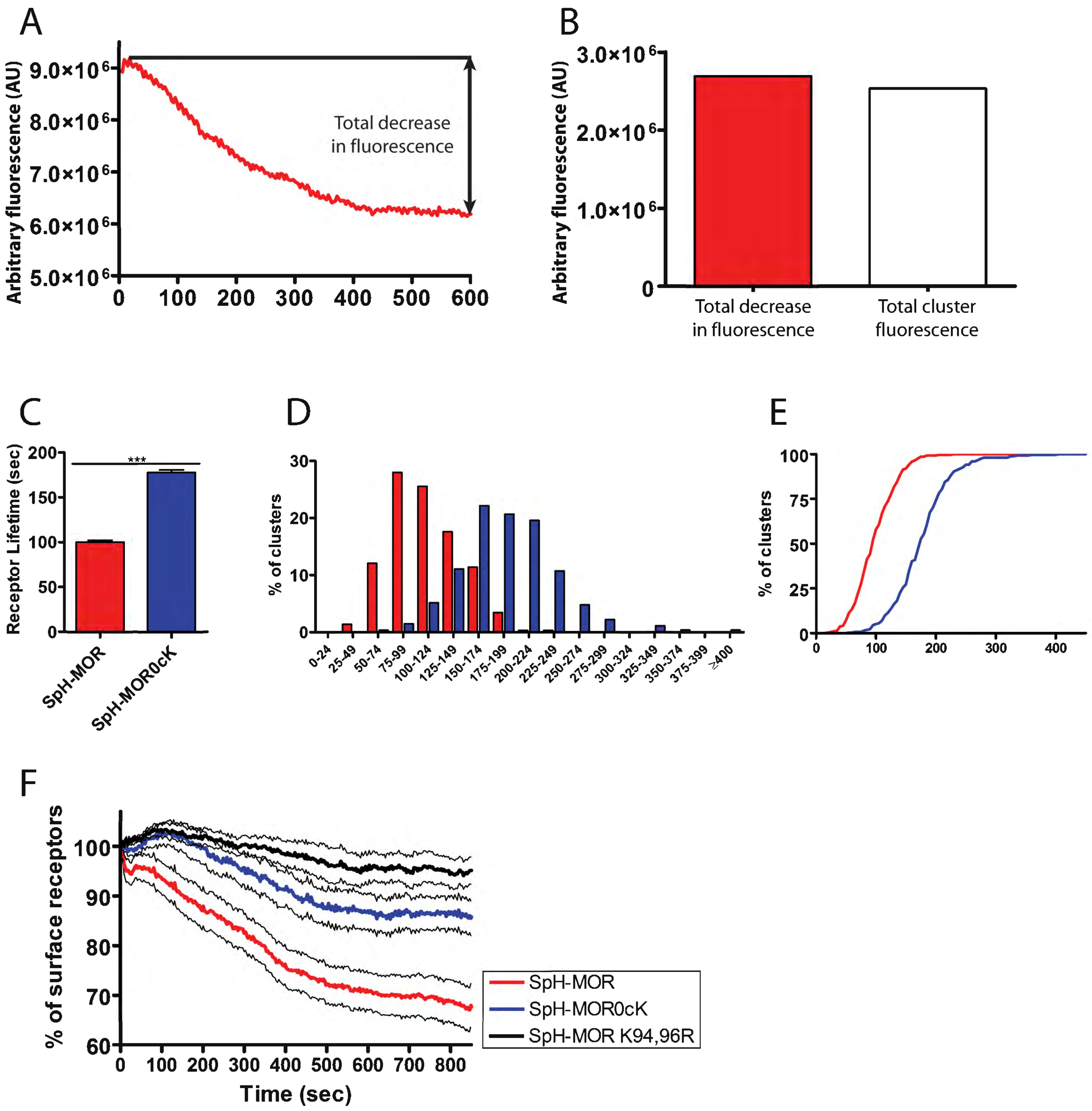
A
Arbitrary fluorescence (AU)
Total decrease
in fluorescence
B
Total decrease
in fluorescence
Total cluster
fluorescence
C
Receptor Lifetime (sec)
***
SpH-MOR
SpH-MOR0cK
D
% of clusters
E
% of clusters
F
% of surface receptors
Time (sec)
SpH-MOR
SpH-MOR0cK
SpH-MOR K94,96R

Figure S3

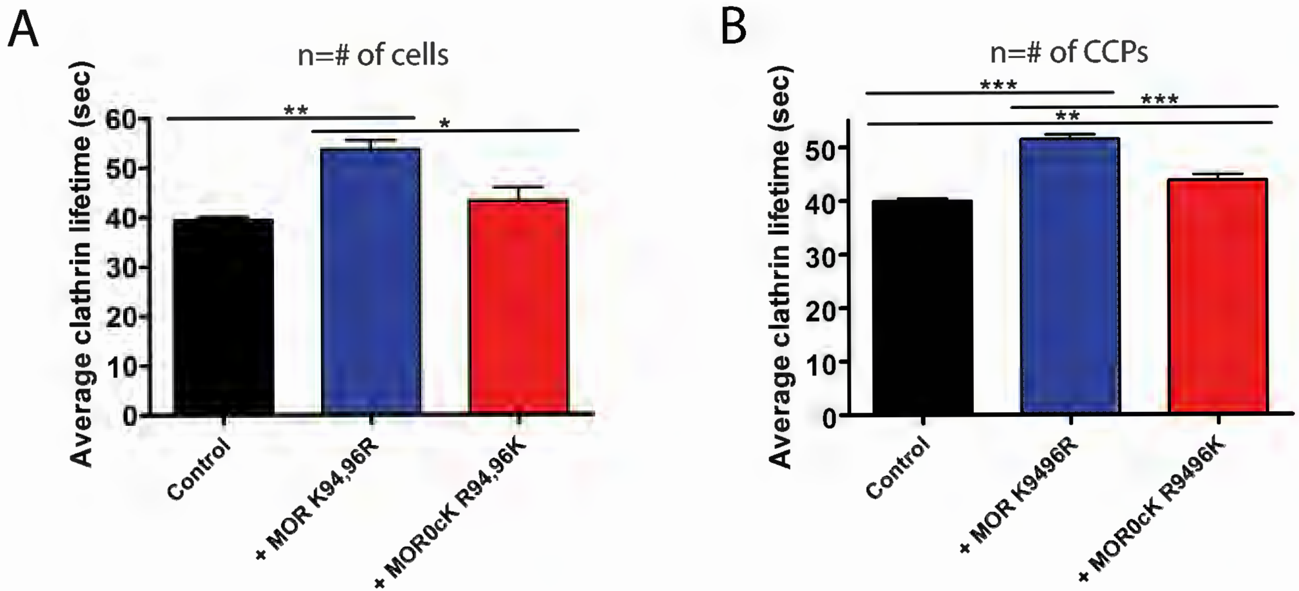
A
n=# of cells
Average clathrin lifetime (sec)
60
50
40
30
20
10
0
**
*
Control
+ MOR K94,96R
+ MOR0cK R94,96K
B
n=# of CCPs
Average clathrin lifetime (sec)
50
40
30
20
10
0
***
***
**
Control
+ MOR K9496R
+ MOR0cK R9496K

Figure S4

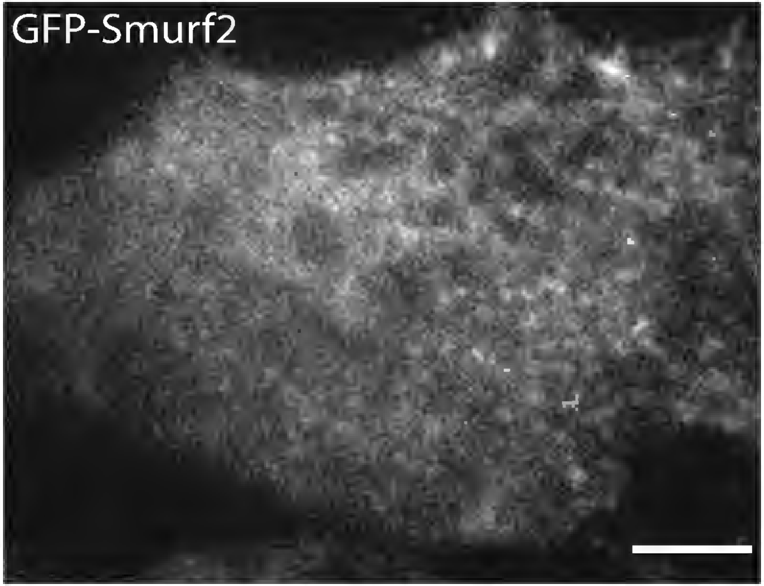

Figure S5

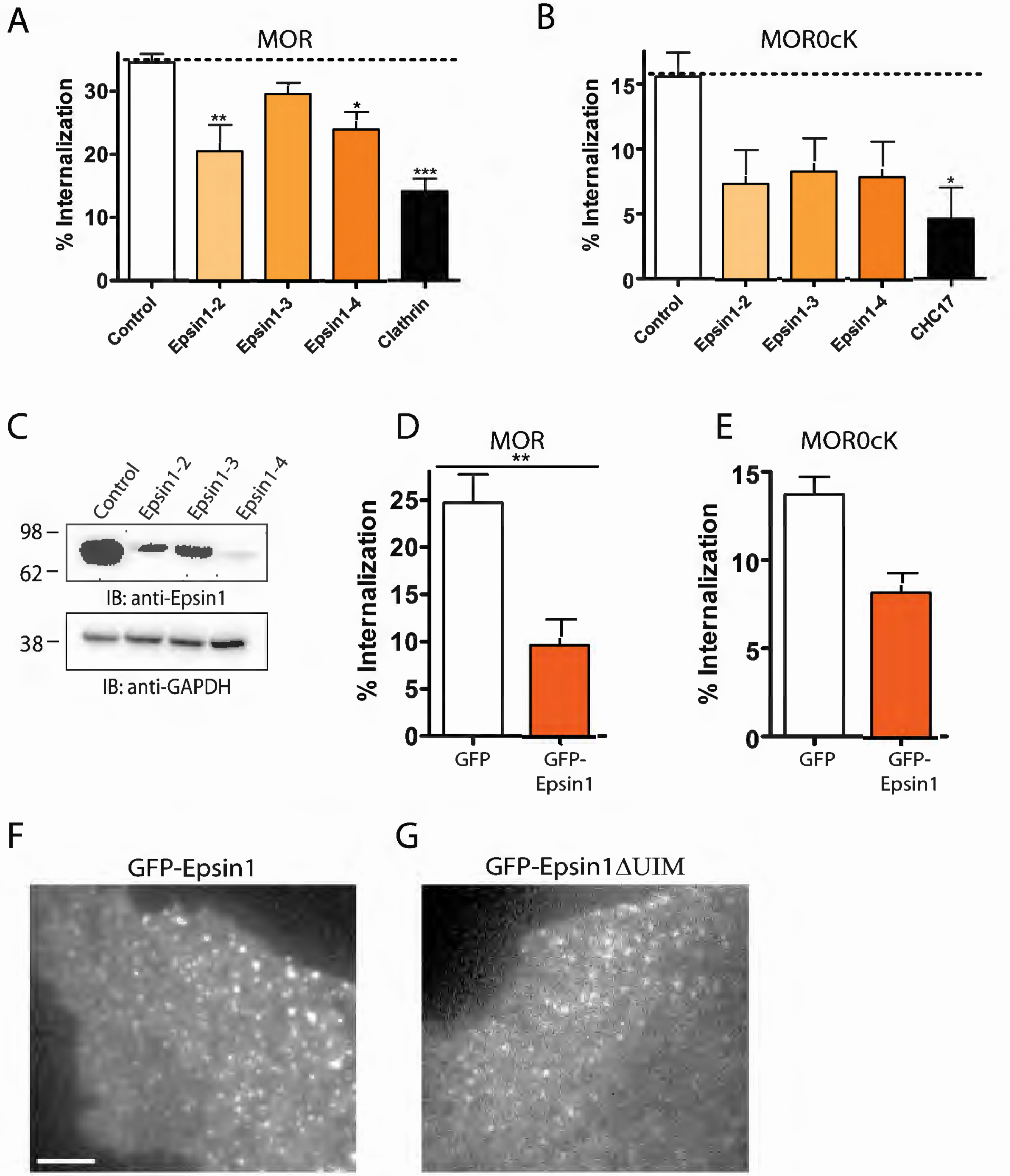
A
MOR
% Internalization
0
10
20
30
**
*
***
Control
Epsin1-2
Epsin1-3
Epsin1-4
Clathrin
B
MOR0cK
% Internalization
0
5
10
15
*
Control
Epsin1-2
Epsin1-3
Epsin1-4
CHC17
C
Control
Epsin1-2
Epsin1-3
Epsin1-4
98
62
IB: anti-Epsin1
38
IB: anti-GAPDH
D
MOR
**
% Internalization
0
5
10
15
20
25
GFP
GFP-Epsin1
E
MOR0cK
% Internalization
0
5
10
15
GFP
GFP-Epsin1
F
GFP-Epsin1
G
GFP-Epsin1ΔUIM

Supplemental Figure Legends

Figure S1. Compiled analysis of the time until appearance of receptor-containing CCPs under different conditions that affect receptor ubiquitination. The time until cluster appearance after agonist addition is measured for F-MOR and F-MOR0cK (A), F-MOR0cK R94,96K and F-MOR K94,96R (B), SpH-MOR and SpH-MOR0cK (C), control or Smurf2 siRNA-transfected F-MOR (D), and GFP- or GFP-Epsin1 Δ UIMs-transfected (E) receptor clusters. The mean time until cluster appearance is depicted by a line for each condition analyzed. P-values: student's t-test; n.s., not significant; ***, $p<0.001$.

Figure S2. The role of receptor ubiquitination on MOR endocytosis measured using SpH-tagged receptors imaged in TIRF-M. (A and B) The total decrease in receptor integrated fluorescence over time approximates the total fluorescence contained within receptor clusters. The total decrease in receptor fluorescence is shown for a representative F-MOR expressing cell treated with agonist at t=0 (A). The total fluorescence of all receptor clusters is measured for the same cell as in E, and is of a similar magnitude to the loss in receptor fluorescence over time (B). (C) The average surface lifetimes of SpH-MOR and SpH-MOR0cK receptor clusters after agonist treatment; MOR n=290 clusters, 8 cells; MOR0cK n=271 clusters, 8 cells. (D) Frequency distribution analysis of SpH-MOR (red) and SpH-MOR0cK (blue) receptor clusters with the specified lifetimes on the cell surface. (E) Cumulative probability analysis of receptor cluster lifetimes. (F) The normalized integrated surface fluorescence of SpH-MOR (red), SpH-MOR0cK(blue), and SpH-MOR K94,96R (black) expressing cells measured every 3 s after agonist addition at time=0 (n=13 cells). The integrated fluorescence value at t=0 is defined as 100%. Error bars indicate standard error of the mean (SEM). P-values: student's t-test; ***, $p<0.001$.

Figure S3. Automated analysis of overall clathrin lifetimes in receptor-expressing cells. (A) The average clathrin lifetimes prior to agonist treatment in cells transiently expressing DsRed-tagged clathrin light chain and pCDNA 3.0 (Control), n=8 cells; F-MOR K94,96R (+ MOR K94,96), n=7cells; or F-MOR0cK R94,96K (+ MOR0cK R94,96K), n= 7cells. (B) The same average clathrin lifetimes from A, where n corresponds to the number of total clusters analyzed. Control n=14013; + MOR K94,96R n=7073; + F-MOR0cK R94,96K n=3629. Error bars indicate standard error of the mean (SEM). P-values: one-way ANOVA, Bonferroni multiple comparison test; *, p<0.05; **, p<0.01; ***, p<0.001.

Figure S4. Smurf2 localizes both diffusely and in CCPs after agonist-induced recruitment. Cells expressing GFP-tagged Smurf2 and F-MOR were imaged live using TIR-FM. Shown is a representative example showing the localization of GFP-Smurf2 in cells treated with agonist for five minutes. Scale bar = 5 μm.

Figure S5. Overexpression or knockdown of Epsin1 inhibits MOR endocytosis irrespective of lysyl-mutation. (A and B) Cells stably-expressing F-MOR (A) or F-MOR0cK (B) were transiently transfected with siRNA duplexes against Epsin1 or CHC17 (as a control) and the percentage of MOR (n≥9) or MOR0cK (n≥4) internalization after 30 minutes agonist treatment was measured using flow cytometric analysis. (C) Verification of Epsin1 knockdown by immunoblotting with an antibody against endogenous Epsin1. Shown is a representative image of three experiments, and equal loading was confirmed by immunoblot detecting GAPDH. (D and E) Cells stably-expressing F-MOR (D) or F-MOR0cK (E) were transiently transfected with either GFP alone or GFP-tagged Epsin1 and the percentage of MOR (n=5) or MOR0cK (n=4) internalization after 30 minutes agonist treatment was measured using flow cytometry. Results were averaged across multiple experiments (shown are mean and SEM). P-values: one-way

ANOVA, Bonferroni multiple comparison test; *, $p<0.05$; **, $p<0.01$, ***, $p<0.001$. (F and G) Cells expressing GFP-tagged Epsin1 and Epsin1ΔUIM and F-MOR were imaged live using TIR-FM. Shown are representative examples showing the localization of GFP-Epsin1 (F) and GFP–Epsin1 ΔUIM (G). Scale bar = 5 μm.

Supplemental Experimental Procedures

Expression constructs and other reagents

The FLAG-tagged MOR construct, DsRed-tagged clathrin light chain, and inactive mutant versions of the E3 ligases Cbl, Mdm2, AIP4, Nedd 4.1 and 4.2, Nedd L1 and 2, WWP1 and 2, and Smurf1 and 2 were previously described (Hislop et al., 2009; Merrifield et al., 2002; Tanowitz and von Zastrow, 2003). FLAG-tagged MOR0cK, MOR0cK R94,96K, MOR0cK R170,181K, MOR0cK R256,265,267K, MOR0cK R340K, and MOR K94,96R were generated by site-directed mutagenesis (QuikChange, Stratagene). Myc-tagged Smurf2 and GFP-tagged inactive mutant version of Smurf2 were generated using PCR and ligation into pcDNA3 and pEGFP-C1, respectively. GFP-tagged Smurf2 was made using PCR and ligation into pEGFP-C1. Epsin1 cDNA was obtained from Open Biosystems and subloned into pEGFP-C1. Epsin1 cDNA was obtained from Open Biosystems and subcloned into pEGFP-C1. GFP-Epsin1ΔUIM was generated using oligonucleotide-directed mutagenesis (QuikChange, Stratagene). Transfections were caaried out using Lipofectamine 2000 or RNAi-max (Invitrogen) for cDNA or siRNA, respectively, according to manufacturer's instructions. Stably-transfected cell lines expressing FLAG-tagged receptors were generated by selection for neomycin resistance with G418 (Geneticin; Invitrogen). Alexa 555-labelled siRNA duplexes against Smurf2 and siRNA duplexes against Epsin1 were obtained from Qiagen and siRNA duplexes against clathrin (CHC 17) were ordered from Qiagen based on an established siRNA sequence (Vassilopoulos et al., 2009). Antibodies used were anti-Smurf2 (Santa Cruz Biotechnology), anti-ubiquitin (P4D1, Santa Cruz Biotechnology), anti-clathrin heavy chain (Santa Cruz Biotechnology), anti-Epsin1 (SantaCruz Biotechnology, R-20 antibody), rabbit anti-FLAG (Sigma), anti-FLAG-M1 (Sigma),

and anti-HA-11 (Covance). Dyngo-4a was obtained from Abcam and used at a final concentration of 30µM.

TIR-FM Imaging

Cells were imaged in Opti-MEM I reduced serum media (UCSF Cell Culture Facility) at 37˚C, maintained with a temperature-controlled stage (Bioscience Tools) and objective warmer (Bioptechs). Cells were imaged with an exposure time of 100ms and an EM gain of 300. Surface FLAG-tagged receptors were labeled with Alexa Fluor 488- or 647-conjugated M1 anti-FLAG antibody for visualization. To visualize endocytic events, cells were treated with 10M DADLE on stage. Bleaching controls were obtained by performing identical experiments in the absence of agonist. For dual imaging experiments, minimal bleed-through between channels was verified by imaging samples labeled only with single fluorophores. Representative live images shown were rendered using Adobe Photoshop software.

Immunoelectron microscopy of plasma membrane sheets

HEK 293 cells stably-expressing F-MOR or F-MOR0cK were grown on coverslips and incubated with 10µM DADLE for 2 minutes. Cells were then washed with cold PBS and placed on ice to prevent further receptor internalization. Cell surface receptors were detected by sequential incubation with 10µg/ml rabbit anti-FLAG IgG (Sigma-Aldrich, MO, USA) and protein A conjugated to 10nm gold particles, after which cells were washed in HEPES buffer (25 mM HEPES, 25 mM KCl, and 2.5 mM Mg acetate, pH 7.0). Plasma membrane sheets were then prepared using a previously described “rip-off” technique (Sanan and Anderson, 1991; Signoret et al., 2005). Briefly, cover slips were inverted on to formvar/carbon/poly-L-lysine coated electron microscopy grids and light pressure applied for 10 sec using a rubber cork. The cover slips were lifted away to rip open adhered cells and leave portions of the dorsal plasma

membrane attached to the grids. The material was briefly washed in HEPES buffer and fixed in 4% glutaraldehyde. Samples were post-fixed by sequential incubation in 1% osmium, 1% tannic acid and 1% uranyl acetate, and then air-dried.

Image Analysis

Image analysis was performed using ImageJ software (Wayne Rasband, National Institutes of Health, Bethesda, MD). For surface lifetime measurements, the time between the appearance and disappearance of clusters was measured as previously described (Puthenveedu and von Zastrow, 2006). Briefly, cluster appearance was identified as the frame where the cluster fluorescence increased above the background fluorescence of an adjacent point of the cell being analyzed. Disappearance of a cluster was determined as the frame where cluster fluorescence decreased to the background fluorescence of a neighboring area of the imaged cell, or when the cluster split off and the subsequent cluster decreased to background levels of fluorescence. Only clusters that clearly appeared and disappeared during image acquisition were included. For cluster intensity measurements, cells were chosen with similar fluorescence values, to ensure equal expression of receptor and clathrin. The mean intensity value of clusters immediately prior to endocytic scission and of an identically-sized background region of the cell at that same frame was quantified. Fluorescence values of clusters were then divided by those of background regions and expressed as “Fold over background” values. For change in intensity over time measurements, ROIs were drawn around individual cells, and the change in integrated intensity was measured and background-corrected. These values were normalized to the intensity of the cell immediately prior to agonist treatment and are represented as “% of initial fluorescence” values.

Detection of ubiquitination by Western blot analysis

Cells stably-expressing FLAG-tagged wildtype or mutant MORs were treated with agonist for

the indicated time points, lysed in 10mM Tris pH 7.4, 1% SDS, and 10mM iodoacetamide supplemented with a standard protease inhibitor mixture (Roche Applied Science), sonicated, and clarified by centrifugation as previously described (Hislop et al., 2011). Samples were incubated overnight with rabbit anti-FLAG antibody (Sigma) and later with protein A/G-agarose beads (Piece), then washed and incubated with SDS sample buffer (Invitrogen) supplemented with DTT. Western immunoblot analysis was performed using anti-HA-11 (Covance) or anti-ubiquitin (Santa Cruz Biotechnology). Blots were probed with anti-ubiquitin or anti-FLAG antibody to verify equal loading and receptor levels.

Automated CCP lifetime analysis

Cells were transiently-transfected with DsRed-tagged clathrin light chain and pCDNA 3.0, F-MOR K94,96R, or F-MOR0cK R94,96K and were treated with agonist while imaging using TIRF-M. Fluorescent particle detection and CCP lifetime tracking was performed on raw image sequences using a previously established Matlab package that is publicly available (Jaqaman et al., 2008; Loerke et al., 2009).

Statistical analysis

Quantitative measurements were averaged across multiple independent experiments, with the number of experiments indicated in the corresponding figure legends. Error bars represent the standard error of the mean quantified after compiling mean determinations across multiple experiments. The statistical significance of the measured differences between conditions were analyzed using the appropriate variations of one- or two-way ANOVA and post-test, specified in figure legends, calculated using Prism 4.0 software (GraphPad Software, Inc). The relative significance of each of the reported differences is indicated by the calculated p values listed in the figure legends and shown graphically in the figures.

Supplemental References

Hislop, J. N., Henry, A. G., Marchese, A., and von Zastrow, M. (2009). Ubiquitination regulates proteolytic processing of G protein-coupled receptors after their sorting to lysosomes. J Biol Chem *284*, 19361-19370.

Hislop, J. N., Henry, A. G., and von Zastrow, M. (2011). Ubiquitination in the first cytoplasmic loop of {micro}-opioid receptors reveals a hierarchical mechanism of lysosomal downregulation. J Biol Chem.

Jaqaman, K., Loerke, D., Mettlen, M., Kuwata, H., Grinstein, S., Schmid, S. L., and Danuser, G. (2008). Robust single-particle tracking in live-cell time-lapse sequences. Nat Methods *5*, 695-702.

Loerke, D., Mettlen, M., Yarar, D., Jaqaman, K., Jaqaman, H., Danuser, G., and Schmid, S. L. (2009). Cargo and dynamin regulate clathrin-coated pit maturation. PLoS Biol *7*, e57.

Merrifield, C. J., Feldman, M. E., Wan, L., and Almers, W. (2002). Imaging actin and dynamin recruitment during invagination of single clathrin-coated pits. Nat Cell Biol *4*, 691-698.

Puthenveedu, M. A., and von Zastrow, M. (2006). Cargo regulates clathrin-coated pit dynamics. Cell *127*, 113-124.

Sanan, D. A., and Anderson, R. G. (1991). Simultaneous visualization of LDL receptor distribution and clathrin lattices on membranes torn from the upper surface of cultured cells. J Histochem Cytochem *39*, 1017-1024.

Signoret, N., Hewlett, L., Wavre, S., Pelchen-Matthews, A., Oppermann, M., and Marsh, M. (2005). Agonist-induced endocytosis of CC chemokine receptor 5 is clathrin dependent. Mol Biol Cell *16*, 902-917.

Tanowitz, M., and von Zastrow, M. (2003). A novel endocytic recycling signal that distinguishes the membrane trafficking of naturally occurring opioid receptors. J Biol Chem *278*, 45978-45986.

Vassilopoulos, S., Esk, C., Hoshino, S., Funke, B. H., Chen, C. Y., Plocik, A. M., Wright, W. E., Kucherlapati, R., and Brodsky, F. M. (2009). A role for the CHC22 clathrin heavy-chain isoform in human glucose metabolism. Science *324*, 1192-1196.
